# BMI and well-being in people of East Asian and European ancestry: a Mendelian randomisation study

**DOI:** 10.1038/s41398-023-02539-7

**Published:** 2023-07-11

**Authors:** Jessica O’Loughlin, Francesco Casanova, Amanda Hughes, Zammy Fairhurst-Hunter, Liming Li, Zhengming Chen, China Kadoorie Biobank Collaborative Group, Jack Bowden, Ed Watkins, Rachel M. Freathy, Laura D. Howe, Robin G. Walters, Jessica Tyrrell

**Affiliations:** 1 College of Biomedical and Clinical Sciences, Faculty of Health and Life Sciences, University of Exeter, Exeter, UK; 2 MRC Integrative Epidemiology Unit (IEU), Population Health Sciences, Bristol Medical School, University of Bristol, Bristol, UK; 3 Clinical Trial Service Unit and Epidemiological Studies Unit (CTSU), Nuffield Department of Population Health, University of Oxford, Oxford, UK; 4 Department of Epidemiology and Biostatistics, School of Public Health, Peking University, Beijing, China; 5 MRC Population Health Research Unit, Nuffield Department of Population Health, University of Oxford, Oxford, UK; 6 Department of Psychology, University of Exeter, Exeter, UK

**Keywords:** Psychiatric disorders, Genomics

## Abstract

Previous studies have linked higher body mass index (BMI) to lower subjective well-being in adult European ancestry populations. However, our understanding of these relationships across different populations is limited. Here, we investigated the association between BMI and well-being in people of (a) East Asian and (b) European ancestry in the China Kadoorie Biobank (CKB) and UK Biobank (UKB), respectively. Mendelian randomisation (MR) methods were used to test the relationship between BMI with (a) health satisfaction and (b) life satisfaction. One-sample MR enabled us to test effects in men and women separately and to test the role of cultural contexts by stratifying our analyses by urban and rural home location in both China and the UK. Further, we implemented a control function method to test the linearity of the BMI-well-being relationship. We found evidence of different associations between BMI and well-being in individuals of East Asian versus European ancestry. For example, a genetically instrumented higher BMI tentatively associated with higher health satisfaction in people of East Asian ancestry, especially in females (ß: 0.041, 95% CI: 0.002, 0.081). In contrast, there was a robust inverse association between higher genetically instrumented BMI and health satisfaction in all European ancestry UKB participants (ß: −0.183, 95% CI: −0.200, −0.165, *P*_difference_ < 1.00E−15). We also showed the importance of considering non-linear relationships in the MR framework by providing evidence of non-linear relationships between BMI and health and life satisfaction. Overall, our study suggests potential setting-specific causality in the relationship between BMI and subjective well-being, with robust differences observed between East Asians and Europeans when considering very similar outcomes. We highlight the importance of (a) considering potential non-linear relationships in causal analyses and (b) testing causal relationships in different populations, as the casual nature of relationships, especially relationships influenced by social processes, may be setting-specific.

## Introduction

Subjective well-being, a measure that seeks to reflect happiness and life satisfaction, is derived from people’s emotional and cognitive evaluations of their quality of life [1, 2]. In 1946, the World Health Organisation (WHO) re-defined “health” as a state of complete physical, mental and social well-being and not merely the absence of disease or infirmity [3]. Here, the concept of health is extended to quality of life with emphasis on a person’s general well-being [4]. The WHO also emphasise the importance of well-being in their definition of “good mental health” [4]. Understanding factors that contribute to lower well-being is crucial to ensure appropriate public health prevention strategies and messaging.

There is extensive evidence linking higher Body Mass Index (BMI) to lower subjective well-being in adult European (EUR) ancestry populations [5–7]. For example, Mendelian randomisation (MR) approaches have provided some evidence for a causal role of higher BMI on lower subjective well-being scores [7, 8]. This association was driven by an effect of BMI on people’s satisfaction with their health, with little evidence of an effect on happiness.

Furthermore, studies have extended these analyses to tease apart the adverse metabolic and other health consequences of high BMI from other factors including psychosocial and cultural influences [8, 9]. MR methods tested the relationship between favourable adiposity (higher BMI but a more favourable metabolic profile) and unfavourable adiposity (higher BMI and a less favourable metabolic profile) with well-being. This provided evidence for a casual role of both higher favourable and unfavourable adiposity on well-being in adult EUR ancestry populations, highlighting the importance of non-metabolic consequences in this relationship, for example, social stigma and perceptions of obesity [8].

Currently, our understanding of the relationship between obesity and well-being across different populations is limited. The social patterning of body weight changes over the course of economic development; as a country’s wealth increases, obesity moves from being more common in higher socioeconomic groups to being associated with socioeconomic disadvantage [10–12]. This is likely to reflect larger body size being regarded as a positive status symbol in some cultures. Additionally, in many low- and middle-income countries, limited access to high-calorie foods may initially serve as a protective factor against obesity [13]. However, as these countries undergo economic development and adopt more Western-style diets, the increased availability and affordability of energy-dense, nutrient-poor foods contribute to a rapid rise in obesity rates [14]. This dietary transition is characterised by a higher consumption of processed foods, added sugars and unhealthy fats [15]. As a result, the burden of obesity and associated non-communicable diseases is shifting towards low- and middle-income countries, posing a significant challenge to their healthcare systems and economies [16]. Observational studies in people of East Asian (EAS) ancestry have shown both high and low BMI to associate with lower subjective well-being [17, 18] but the study designs have not enabled causal inferences to be made.

Ideally, evidence of causal effects comes from well-conducted randomised control trials (RCTs). However large-scale RCTs cannot always be performed because they can be costly, impractical or even unethical. One of the alternatives is to perform MR analyses that are similar to RCTs in terms of study design. MR uses genetic variation as a natural experiment to investigate the causal relations between potentially modifiable risk factors and health outcomes in observational data [19]. To date, no studies have used genetic techniques to test the causal role of BMI on well-being in adults of EAS ancestry. The identification of causal associations in different sociocultural contexts will provide important information about the complex and potentially setting-specific relationships between obesity and well-being, thereby informing global decisions on medical management and public health strategies.

Traditional linear models assume that the relationship between BMI and well-being is linear, but recent research has challenged this assumption. Several studies have suggested that the relationship between BMI and well-being may follow a non-linear pattern, with both low and high BMI levels being associated with decreased well-being [20–22]. Traditional linear models may fail to capture the complexity of this relationship, leading to biased estimates and incorrect conclusions. Therefore, it is crucial to investigate non-linear relationships in observational studies of BMI and well-being.

By uncovering the true nature of the relationship between BMI and well-being, researchers can identify at-risk populations and tailor interventions accordingly. For instance, a non-linear relationship could indicate that interventions should target individuals with specific BMI ranges to improve their well-being, rather than a one-size-fits-all approach. Moreover, investigating non-linear relationships could help us understand the complex interplay between these variables, leading to more accurate estimates and better-targeted interventions.

Here, we utilise MR methods, to provide important information about the relationship between BMI and well-being. Using data from the China Kadoorie Biobank (CKB) and the UK Biobank (UKB) to directly compare the relationship between BMI and well-being in East Asian and European populations, we (1) investigate the association between BMI and well-being in people of (a) EAS and (b) EUR ancestry; (2) test effects in men and women separately; (3) further test the role of cultural contexts by stratifying our analyses by urban and rural home location in both China and the UK; and (4) test the linearity of the BMI-well-being relationship. We show that the relationship between BMI and well-being differs across different contexts – “setting-specific causality” – providing evidence that population-level public health approaches to obesity and well-being may need to take into account the cultural and environmental characteristics of the target population.

## Methods

### China Kadoorie Biobank

The CKB (www.ckbiobank.org) is a study of 512,891 adults aged between 30 and 79 years at recruitment, with the baseline survey occurring between 2004–2008. The CKB and data collection has been described in detail elsewhere [23], but briefly the baseline survey took place in 10 geographically defined regions in China (5 urban, 5 rural) and detailed questionnaire data, physical measurements and blood samples were taken from all participants. The participants agreed to have their health followed via linkages with clinical registries and health insurance databases. All analyses were conducted under project 2019–0003 as approved by the CKB Research Committee, using dataset DAR-2021-00041 from data release 17.02.

### UK Biobank

The UKB is a health resource with extensive phenotypic and genetic data available for over 500,000 participants, who were aged between 40 and 70 at recruitment (from 2006 to 2010). Participants were recruited from across the UK and attended one of 22 centres in England, Scotland and Wales, to provide detailed sociodemographic, health and anthropometric data as well as providing blood and urine samples for subsequent analyses. Participants consented to having their health followed and many have subsequently participated in further monitoring or completed additional questionnaires. The cohort is described in detail elsewhere, with information on ethics and recruitment [24]. This research has been conducted using the UK Biobank resource under application number 9072.

### Genetic variants

#### China Kadoorie Biobank

Genotyping in the CKB has been previously described [25]. Briefly, a total of 102,783 participants were genotyped using two custom-designed Affymetrix Axiom arrays, including up to 803,000 variants, optimised for genome-wide coverage in Chinese populations. Stringent QC resulted in genotypes for 532,415 variants present on both array versions. Genotypes were imputed to the 1000 Genomes Phase 3 reference (EAS MAF > 0) using SHAPEIT version 3 and IMPUTE version 4.

Genotype data were available for 100,574 individuals whose samples passed QC (call rate >99.97% across all variants).

### BMI variants selected

The BMI variants that reach *P* < 1 × 10^−8^ in independent genome-wide association studies in European ancestries were extracted from the imputed CKB data (Supplementary Table 1) [26]. Variants were then coded based on the trait-increasing allele in CKB; this was assessed in each sex by regressing rank-inverse normal transformed (RINT) BMI against the corresponding set of variants in a univariate multiple regression model. When the trait-increasing allele differed between men and women, the sex in which the effect size was largest was used to code the effect allele in both sexes. Block jack-knifing was then used to weight the variants. In the unrelated subset of CKB participants with valid genetic data (*n* = 72,698), the variants were regressed against BMI in men and women separately, with adjustment for 12 national principal components (PCs). The resulting effect sizes were used to weight the variants in the related individuals.

To weight the variants in the unrelated individuals, the unrelated subset was split into 100 blocks. Each block was then iteratively removed from the univariate multiple regression model and estimated effect sizes applied as weights to the individuals excluded from the regression. These weights were used to create a genetic risk score (GRS) in CKB (Eq. 1). Multiallelic variants or variants with a MAF < 0.01 in CKB were excluded from GRS generation.

Within CKB, the BMI GRS was robustly associated with BMI, explaining 1.5% (*F* = 637.87) and 3.1% (*F* = 1815.94) of the variance in males and females, respectively.

#### UK Biobank

Genotyping in the UKB has been described in more detail elsewhere [27]. Briefly, precisely imputed (INFO score > 0.9) genetic variants were selected from the UK Biobank’s imputation data (released in 2017). Genome-wide genotyping was performed on 451,025 individuals using the UK Biobank Axiom Array, and on ~50,000 individuals using the UK Biobank BiLEVE array. The two SNP arrays were very similar with over 95% common marker content. PCA was performed to determine population stratification. Principal components (PCs) were generated in the 1000 Genomes Cohort using high-confidence SNPs to obtain their individual loadings. These loadings were then used to project all the UK Biobank samples into the same principal component space, and individuals were then clustered using PCs 1–4. Participants were removed if they had subsequently withdrawn from the study (*n* = 111) or if they were sex mismatches (*n* = 348; self-reported sex did not match genetic sex).

A subset of unrelated individuals (*n* = 379,768) was defined from the 451,025 individuals of white EUR ancestry, and the KING Kinship matrix was used to separate out related individuals (up to third degree). An optimal list of unrelated individuals was generated to allow maximum numbers of individuals to be included. Ancestral PCs were then generated within these identified individuals for use in subsequent analyses.

### BMI variants selected

Genetic variants associated with BMI at genome-wide significance (*P* < 5 × 10^−8^) in the GIANT consortium of up to 339,224 people of EUR ancestry were selected (Supplementary Table 2) [28]. Independent loci were defined by a clumping analysis on a European-only GWAS-based meta-analysis, using LD *r*2 > 0.1 and a distance criterion of ±500 kb surrounding each genome-wide significant peak (*P* < 5 × 10^−8^) [28]. Here, we used an alternative set of SNPs to those used in the CKB GRS as the discovery sample did not include the UK Biobank and therefore avoids over-fitting.

The variants were recoded as 0, 1 and 2 according to the number of BMI-increasing alleles. A weighted GRS was created using BMI variants (Eq. 1). Each variant was weighted by its effect size (β-coefficient) obtained from the primary GWAS that did not include any data from the UK Biobank [28].

Within UKB, the variants included explained 1.8% (*F* = 3230.42) and 1.5% (*F* = 3114.02) of the variance in BMI in males and females, respectively.

### Genetic risk scores

Genome wide significant variants were used to create a weighted GRS . Firstly, variants were recoded to represent the risk-increasing alleles. Each variant was then weighted by its effect size (Eq. 1).

Equation 1 Weighted Genetic Risk Score

$$GRS_w = \beta _1d_1 + \beta _2d_2 + \ldots + \beta _nd_n = \mathop {\sum}\limits_{i = 1}^n {\beta _id_i}$$

$$GRS_s = \frac{{n \times GRS_w}}{{\mathop {\sum}\nolimits_{i = 1}^n {\beta _i} }}$$

Where *β*_*i*_ represents the effect size and *d*_*i*_ represents the effect allele dosages for variant *i* of *n*, and *GRS*_*w*_ and *GRS*_*s*_ represent the weighted and standardised GRSs, respectively.

### Exposure and outcome measures

Exposure: body mass index (BMI)

In both CKB and UKB, BMI was calculated as weight (kg) divided by the square of standing height (m). BMI was available for 100,574 and 379,708 individuals with valid genetic data in the CKB and UKB, respectively.

Outcome: well-being

#### Health satisfaction

In CKB, participants were asked “How is your current self-rated health status?" with the options to respond “Poor”, “Fair”, “Good” or “Excellent” (Supplementary Table 3). Similarly, in UKB all participants were asked “In general how would you rate your overall health” with the options to respond “Poor”, “Fair”, “Good” or “Excellent”. We recoded these variables 0 to 3 with 3 representing “Excellent”.

Amongst genotyped participants, 100,574 and 379,708 had information on health satisfaction in CKB and UKB, respectively.

#### Life satisfaction

In CKB, participants were asked “In general, how satisfied are you with your life?" with the options to respond “Very unsatisfied”, “Unsatisfied”, “Neither satisfied nor dissatisfied”, “Satisfied” or “Very satisfied”. Due to low numbers (0.3%) of individuals reporting to be “Very unsatisfied”, we combined this with the “Unsatisfied” category and recoded this variable 0–3 with 3 representing “Very satisfied” (Supplementary Table 4).

In UKB, all participants completing the Mental Health Questionnaire (MHQ) were asked: “In general how happy are you?” (Data field 20458) with the options to respond, “Extremely unhappy”, “Very unhappy”, “Moderately unhappy”, “Moderately happy”, “Very happy”, “Extremely happy”, “Do not know” and “Prefer not to answer”. We recoded this variable with 0 to 5 with 5 representing “Extremely happy”. Participants who preferred not to answer or did not know were set to missing.

Amongst genotyped participants, 100,574 and 130,298 had information on life satisfaction in CKB and UKB, respectively.

### Data analysis

#### Observational associations

BMI was regressed against measures of well-being using linear models, which were adjusted for age at baseline, sex, and region (CKB only), centre (UKB only) and then further adjusted for smoking status, alcohol consumption and measures of socioeconomic status (SES, Supplementary Methods).

#### One-sample Mendelian randomisation

We undertook 1-sample MR analyses to test the causal relationship between BMI and well-being in people of EAS and EUR ancestry.

We employed the two-stage least-squares regression estimator method which uses predicted levels of BMI per genotype and regresses the well-being outcome against these predicted values [29]. First, we calculated the association between the BMI GRS and BMI. These predicted values were then used as the independent variable and well-being as the dependent variable in a linear regression model. In both stages we adjusted for age, regional principal components, and array version.

Results from MR analyses may represent a valid causal effect estimate under the condition of four core assumptions [19, 30]:

1. The genetic instrument needs to robustly associate with the exposure (‘relevance’);
2. There should be no joint causal influence affecting the exposure instrument and the outcome (‘independence’);
3. The instrument must not affect the outcome through any mechanism other than through the exposure (‘exclusion restriction’).
4. The true relationship between the exposure and outcome in each specific analysis is correctly modelled.

An example where assumption 4 would be violated is if a linear relationship between the exposure and outcome in the TSLS model were assumed, implying a constant causal effect across all levels of the exposure, but in fact the true relationship was non-linear. We aimed to test the linearity assumption by additionally fitting quadratic causal effect models (see below).

In CKB, the analyses were performed stratified by sex and recruitment region, using region-specific principal components. The resulting estimates and standard errors were then meta-analysed using a fixed effects model to provide estimates for urban and rural dwellers and for all individuals. The heterogeneity of the estimates was assessed using the *I*^2^ statistic. For UKB, the analyses were run in all individuals and there was no need for meta-analysis.

#### Differences between CKB and UKB estimates

To test the hypothesis that the effects of BMI on well-being may differ in individuals of EAS and EUR ancestry we compared our genetic estimates using the Fisher’s *z*-score method (Eq. 1) [31].

1 $$z = \frac{{\beta _1 - \beta _2}}{{\sqrt {SE_1^2 + SE_2^2} }}$$

#### Sensitivity analyses

We repeated the genetic analyses for life satisfaction in CKB excluding the Hunan region, as the prevalence of individuals reporting to be “unsatisfied” in this region was <1% in both males and females (Supplementary Table 4).

#### Non-linear analyses

To test whether the relationship between BMI and well-being outcomes was non-linear, we compared linear, quadratic (U-shaped) and fractional polynomial (FP) models. The quadratic association between BMI status and health and life satisfaction was tested by regressing well-being outcomes on linear and quadratic terms of BMI.

FP modelling is a powerful tool to detect non-linear associations [32–34]. FPs are described in more detail elsewhere [35]. Briefly, there are two classes of FP: First degree (FP1) and second degree (FP2) FPs. FP1 performs eight tests to detect whether the fit is improved by a power transformation of the variable *X, Xp* where *P* is chosen from *S* = (–2, –1, –0.5, 0, 0.5, 1, 2, 3). FP with value of *P* = 1 is synonymous with a linear regression. FP2 is an extension to *β*_1_*Xp*_1_ + *β*_2_*Xp*_2_ which compares 36 different power combinations, with *p*_1_ = 1, *p*_2_ = 2 equivalent to quadratic regression.

Linear and non-linear models were compared based on the Akaike information criterion (AIC), and the model with an AIC value smaller than the other model was considered to be a better fit [36].

#### Non-linear Mendelian randomisation

We performed non-linear MR of the relationship between BMI and measures of subjective well-being in the CKB and UKB using a control function method [37]. In the first stage, we regressed the instrumental variable (BMI GRS) on the exposure (BMI) whilst accounting for covariates. In the second stage, the outcome (well-being) was directly regressed on the exposure (BMI) including both linear and quadratic terms adjusting for covariates and the residuals of the first stage regression, in order to account for unmeasured confounding. The coefficients of the second stage were taken as the control function estimates. We compared the CKB and UKB non-linear estimates using Fisher’s *z*-score method (Eq. 1).

## Results

The demographics of individuals with valid genetic data in CKB and UKB are shown in Table 1. Generally, individuals in the CKB had a lower BMI, were younger, included a higher proportion of females, and a lower proportion of participants were living in urban regions when compared to participants in the UKB (Table 1).

**Table 1:** The demographics and lifestyle characteristics of participants in the CKB and UKB with valid genetic data and measured BMI.

Demographic	CKB	UKB	*P*^a^
*N*	100,574	379,708	–
Mean age at recruitment (SD)	53.7 (11.0)	57.2 (8.0)	<1.00E-15
Female, *N* (%)	57,573 (57.2)	204,736 (53.9)	<1.00E-15
Lives in urban region, *N* (%)	43,939 (43.7)	320,127 (85.1)	<1.00E-15
Mean BMI (SD)	23.7 (3.5)	27.4 (4.8)	<1.00E-15
Mean WHR (SD)	0.88 (0.1)	0.87 (0.1)	–
Sleep hours (SD)	7.37 (1.4)	7.33 (0.95)	8.22E-12
Mean MET hours (physical activity metric) (SD)	6.03 (3.5)	7.40 (1.1)	<1.00E-15
Smoking status, *N* (%)			<1.00E-15
Never smoker	60,314 (60.0)	204,218 (53.8)	–
Former smoker	6921 (6.9)	134,423 (35.4)	–
Current smoker	27,670 (27.5)	35,940 (9.5)	–
Occasional smoker (CKB)/ Missing (UKB)	5669 (5.6)	5127 (1.4)	–
Highest education, *N* (%)			<1.00E-15
No formal school	20,141 (20.0)	63,515 (16.7)	–
University	2474 (2.5)	180,127 (47.4)	–
Other	77,959 (77.5)	132,445 (34.9)	–
Missing	0 (0)	3621 (1.0)	–
Unemployed *N* (%)	2901 (2.9)	6182 (1.6)	<1.00E-15
Has own home *N* (%yes)	42,883 (42.6)	340,318 (91.7)	<1.00E-15
Health satisfaction *N* (%)	–	–	<1.00E-15
Excellent	17,187 (17.1)	64,053 (16.9)	–
Good	27,187 (27.0)	202,717 (58.4)	–
Fair	44,584 (44.3)	77,460 (20.5)	–
Poor	11,616 (11.6)	16,046 (4.2)	–
Anxiety *N* (%)	682 (0.7)	6163 (7.5)	<1.00E-15
Depressive symptoms *N* (%)	3398 (3.4)	41,644 (14.4)	<1.00E-15
Major depression *N* (%)	760 (0.8)	29,594 (23.8)	<1.00E-15

^a^*P* Comparison of people of East Asian (CKB) and European (UKB) ancestry.

### Health satisfaction

#### Higher BMI was differentially associated with health satisfaction in CKB and UKB using both observational and genetic models

**Fig. 1:**
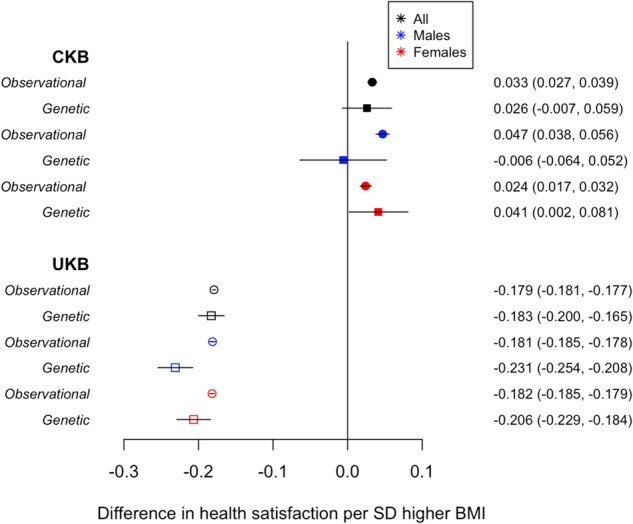
Health satisfaction and BMI in UK Biobank and China Kadoorie Biobank. The observational and 1-sample genetic associations between BMI and health satisfaction in all individuals in the CKB and UKB, stratified by sex.

Observationally, within CKB, a 1-SD higher BMI (3.50 kg/m^2^) was associated with higher health satisfaction (0.033, [0.027, 0.039]) in all individuals (Table 2 and Fig. 1). Estimates were consistent in males (0.047, [0.038, 0.056]) and females (0.024, [0.017, 0.032]; Table 2 and Fig. 1). In contrast, a 1-SD higher BMI (4.80 kg/m^2^) was associated with lower health satisfaction (−0.179, [−0.181, −0.177]) in all individuals in the UKB. Effect estimates were consistent when stratifying by sex (Table 2 and Fig. 1). Adjusting the observational analyses for smoking status, alcohol consumption and measures of SES (Supplementary Methods) slightly attenuated the effect estimates toward the null (Supplementary Table 5).

**Table 2:** The observational and genetic associations between BMI and health satisfaction in the CKB and UKB stratified by sex and urban and rural regions.

			Observational	Genetic		
Study	Strata	Region	Beta (95% CI) per SD higher BMI	*P*^a^	Beta (95% CI) per SD higher BMI	*P*^b^
CKB	All	Both	0.033 (0.027, 0.039)	<1.00E-15	0.026 (−0.007, 0.059)	0.12
	Male	Both	0.047 (0.038, 0.056)	<1.00E-15	−0.006 (−0.064, 0.052)	0.83
	Female	Both	0.024 (0.017, 0.032)	1.10E-10	0.041 (0.002, 0.081)	0.04
	All	Urban only	0.020 (0.011, 0.030)	1.40E-05	0.022 (−0.031, 0.076)	0.41
	Male	Urban only	0.030 (0.015, 0.044)	7.10E-05	−0.004 (−0.100, 0.091)	0.93
	Female	Urban only	0.016 (0.004, 0.028)	0.01	0.035 (−0.030, 0.099)	0.30
	All	Rural only	0.034 (0.027, 0.041)	<1.00E-15	0.028 (−0.013, 0.070)	0.18
	Male	Rural only	0.051 (0.040, 0.063)	<1.00E-15	−0.008 (−0.080, 0.065)	0.84
	Female	Rural only	0.025 (0.015, 0.034)	3.70E-07	0.045 (−0.005, 0.095)	0.08
UKB	All	Both	−0.179 (−0.181, −0.177)	<1.00E-15	−0.183 (−0.200, −0.165)	<1.00E-15
	Male	Both	−0.181 (−0.185, −0.178)	<1.00E-15	−0.231 (−0.254, −0.208)	<1.00E-15
	Female	Both	−0.182 (−0.185, −0.179)	<1.00E-15	−0.206 (−0.229, −0.184)	<1.00E-15
	All	Urban only	−0.178 (−0.181, −0.176)	<1.00E-15	−0.184 (−0.202, −0.165)	<1.00E-15
	Male	Urban only	−0.180 (−0.184, −0.176)	<1.00E-15	−0.234 (−0.260, −0.209)	<1.00E-15
	Female	Urban only	−0.181 (−0.184, −0.178)	<1.00E-15	−0.203 (−0.228, −0.179)	<1.00E-15
	All	Rural only	−0.179 (−0.185, −0.173)	<1.00E-15	−0.167 (−0.213, −0.122)	6.40E-13
	Male	Rural only	−0.185 (−0.194, −0.176)	<1.00E-15	−0.173 (−0.234, −0.113)	1.90E-08
	Female	Rural only	−0.178 (−0.185, −0.170)	<1.00E-15	−0.177 (−0.241, −0.114)	4.20E-08

^a^*P* adjusted for age, region (CKB only), centre (UKB only) and sex.

^b^*P* adjusted for age, region (CKB only), centre (UKB only), sex and principal components.

Genetics provided some evidence for population-specific causality. One-sample MR, in 100,574 East Asian ancestry individuals, provided tentative evidence for a causal role of higher BMI in health satisfaction in females (0.041, [0.002, 0.081]), consistent with the observational results (Table 2 and Fig. 1). By contrast, in males there was no evidence of an association (−0.006, [−0.064, 0.052]). Thus, for meta-analysis across all individuals, a genetically determined 1-SD higher BMI was not significantly associated with higher health satisfaction (0.026, [−0.007, 0.059]), although in each case the genetic association was consistent with that from the observational analysis.

In people of EUR ancestry, 1-sample MR methods provided strong evidence for an inverse causal association between higher BMI and health satisfaction, consistent with the observational analyses. A 1-SD higher BMI was associated with lower health satisfaction in all individuals (−0.183, [−0.200, −0.165]) and in males (−0.231, [−0.254, −0.208]) and females (−0.206, [−0.229, −0.184]), respectively (Table 2 and Fig. 1).

The effect estimates, when using 1-sample MR methods, were substantially different when comparing individuals of East Asian and European ancestry (*P*_diff_<1.00E-15, Supplementary Table 6). Significant differences were also observed in sex stratified analyses (*P*_diff males_ = 1.75E-12 and *P*_diff females_ = 1.24E-04, Supplementary Table 6).

#### Recruitment region within China influenced our findings, whereas higher BMI was consistently associated with lower health satisfaction in UKB

Observationally, higher BMI was associated with higher health satisfaction in all individuals of EAS ancestry living in either urban or rural regions of China (Table 2), with consistent estimates in males and females (Table 2). However, when further adjusting for smoking status, alcohol consumption and SES, in males and females from urban regions the confidence intervals crossed the null (Supplementary Table 5). By contrast, observationally higher BMI was robustly associated with lower health satisfaction in urban and rural dwelling EUR ancestry populations (Table 2).

**Fig. 2:**
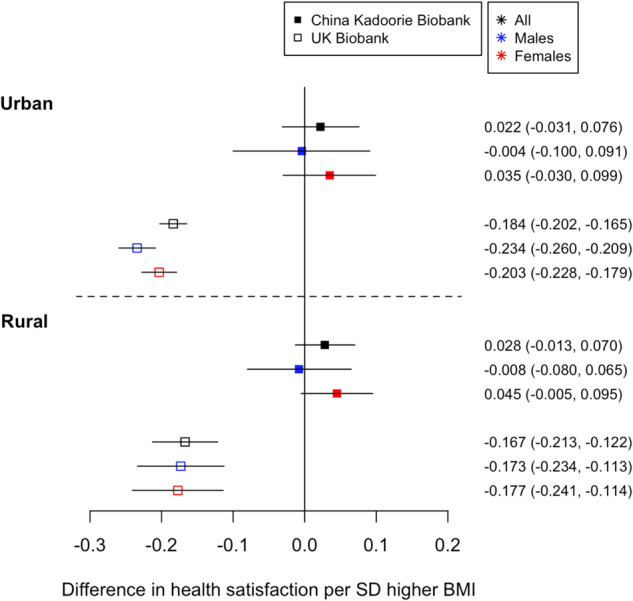
Health satisfaction and BMI stratified by rural and urban home location. The 1-sample genetic associations between BMI and health satisfaction in all individuals in the CKB and UKB stratified by sex and urban versus rural dwelling.

When stratifying by urban and rural regions, there was no clear causal evidence within people of EAS ancestry, although the effect estimates were unchanged (Table 2 and Fig. 2). Across the 10 CKB regions we observed high heterogeneity between the estimates for all participants and for females (*P*_heterogeneity_ < 1.00E-15 Supplementary Table 7 and Supplementary Fig. 1). By contrast, genetically instrumented BMI provided strong evidence that higher BMI lowered health satisfaction in EUR ancestry individuals living in both urban and rural regions of the UKB (Table 2 and Fig. 2). For example, a 1-SD higher BMI was associated with lower health satisfaction (−0.184, [−0.202, −0.165]) in all individuals living in urban areas.

For participants living in both urban and rural environments, comparison of CKB and UKB estimates provided strong evidence for a difference in the relationship between BMI and health satisfaction in individuals of East Asian and European ancestry (*P*_urban_ = 2.79E-04, *P*_rural_ = 5.37E-10; Supplementary Table 6).

### Life satisfaction

#### Limited evidence for an association between BMI and life satisfaction

Observationally, a 1-SD higher BMI was associated with higher life satisfaction (0.058, [0.053, 0.063]) in all individuals of EAS ancestry in the CKB. Estimates were consistent in (a) males and females (Table 3 and Supplementary Fig. 2), (b) urban versus rural dwellers (Table 3), and (c) when further adjusting for SES, alcohol consumption and smoking status (Supplementary Table 5).

**Table 3:** The observational and genetic associations between BMI and life satisfaction in the CKB and UKB stratified by sex and urban and rural regions.

			Observational		Genetic	
Study	Strata	Region	Beta (95% CI) per SD higher BMI	*P*^a^	Beta (95% CI) per SD higher BMI	*P*^b^
CKB	All	Both	0.058 (0.053, 0.063)	<1.00E-15	−0.028 (−0.055, −0.002)	0.04
	Male	Both	0.054 (0.047, 0.062)	<1.00E-15	−0.077 (−0.123, −0.030)	1.00E-03
	Female	Both	0.062 (0.056, 0.069)	<1.00E-15	−0.004 (−0.037, 0.028)	0.80
	All	Urban only	0.048 (0.041, 0.055)	<1.00E-15	0.006 (−0.036, 0.048)	0.78
	Male	Urban only	0.059 (0.048, 0.070)	<1.00E-15	−0.033 (−0.109, 0.042)	0.39
	Female	Urban only	0.043 (0.034, 0.052)	<1.00E-15	0.024 (−0.027, 0.075)	0.36
	All	Rural only	0.066 (0.059, 0.072)	<1.00E-15	−0.051 (−0.085, −0.016)	4.00E-03
	Male	Rural only	0.072 (0.062, 0.081)	<1.00E-15	−0.103 (−0.162, −0.044)	1.00E-03
	Female	Rural only	0.063 (0.055, 0.072)	<1.00E-15	−0.023 (−0.066, 0.019)	0.28
UKB	All	Both	−0.019 (−0.023, −0.014)	<1.00E-15	−0.019 (−0.054, 0.016)	0.28
	Male	Both	−0.002 (−0.008, 0.005)	0.65	−0.003 (−0.049, 0.043)	0.90
	Female	Both	−0.029 (−0.035, −0.023)	<1.00E-15	−0.030 (−0.073, 0.014)	0.19
	All	Urban only	−0.020 (−0.025, −0.015)	2.3E-15	−0.027 (−0.066, 0.011)	0.16
	Male	Urban only	−0.005 (−0.013, 0.002)	0.17	−0.003 (−0.054, 0.048)	0.90
	Female	Urban only	−0.028 (−0.035, −0.022)	<1.00E-15	−0.031 (−0.079, 0.016)	0.19
	All	Rural only	−0.013 (−0.024, −0.002)	0.02	0.030 (−0.058, 0.119)	0.50
	Male	Rural only	0.015 (−0.002, 0.032)	0.08	0.026 (−0.085, 0.137)	0.64
	Female	Rural only	−0.030 (−0.045, −0.015)	6.80E-05	0.010 (−0.111, 0.130)	0.88

^a^*P* adjusted for age, region (CKB only), centre (UKB only) and sex.

^b^*P* adjusted for age, region (CKB only), centre (UKB only), sex, and principal components.

By contrast, observationally a 1-SD higher BMI was associated with lower life satisfaction (−0.019, [−0.023, −0.014]) in all UKB individuals. Effect estimates were consistent urban versus rural dwellers and in females, but in males the observed effect was very small and confidence intervals crossed the null (Table 3 and Supplementary Fig. 2).

One-sample MR in individuals of EAS ancestry provided some evidence for an inverse association between BMI and life satisfaction in males (−0.077, [−0.123, −0.030]); Table 3 and Supplementary Fig. 2), but the association was null in females. Stratifying by urban versus rural home location in CKB suggested the relationship in males was stronger in those from rural regions (−0.103, [−0.162, −0.044]) (Table 3 and Supplementary Fig. 3). However, we observed high heterogeneity across the different regions in CKB (Supplementary Table 7 and Supplementary Fig. 4).

In the UK Biobank, one-sample MR in EUR individuals provided no evidence for a causal role of higher BMI on life satisfaction in all individuals, nor when stratifying by sex and/or home location (Table 3 and Supplementary Figs. 2 and 3).

When comparing CKB and UKB estimates we found only a nominally significant difference in the relationship between BMI and life satisfaction in rural males only (*P*_difference_ = 0.04, Supplementary Table 8).

#### Sensitivity analyses

We repeated our genetic analyses for life satisfaction in CKB excluding Hunan as the prevalence of individuals reporting to be “unsatisfied” in this region was <1% in both males and females. Excluding Hunan lowered the heterogeneity of our estimates (Supplementary Table 7), and in our one-sample analyses the male estimate was no longer significant (Supplementary Table 9 and Supplementary Figs. 5–6).

### Non-linear analyses

Observationally, using quadratic and fractional polynomial (FP) models, we found evidence for a non-linear relationship between BMI and health satisfaction in all individuals from both the CKB and UKB (Supplementary Table 10). The FP2 model demonstrated the lower AIC value for all analyses in both the CKB and UKB, including when stratifying by urban versus rural dwelling and/or sex. Some of the FP2 models were collapsible into FP1 models due to having the same p1 and p2 values, however, they still differed from the FP1 model and showed improved model fit. The selected FP2 models differed between CKB and UKB suggesting the shape of the non-linear association may be different in people of East Asian and European ancestry (Supplementary Table 10). Further, the FP2 models differed by sex and region within the CKB which may suggest differences in the non-linear associations within China (Supplementary Table 10).

Both quadratic and fractional polynomial models provided evidence for a non-linear relationship between BMI and life satisfaction in the CKB and UKB (Supplementary Table 11). The FP2 model demonstrated the lowest AIC value in all individuals from the CKB and UKB and when stratifying by sex. In rural regions of the CKB, the FP1 model demonstrated a better fit (Supplementary Table 11). Additionally, despite the lower AIC value for the FP2 model in most cases, there were specific subsets where the FP1 and FP2 models yielded equivalent results. Specifically, in urban females from the CKB dataset and rural males from the UKB dataset (Supplementary Table 11).

#### Non-linear MR

**Fig. 3:**
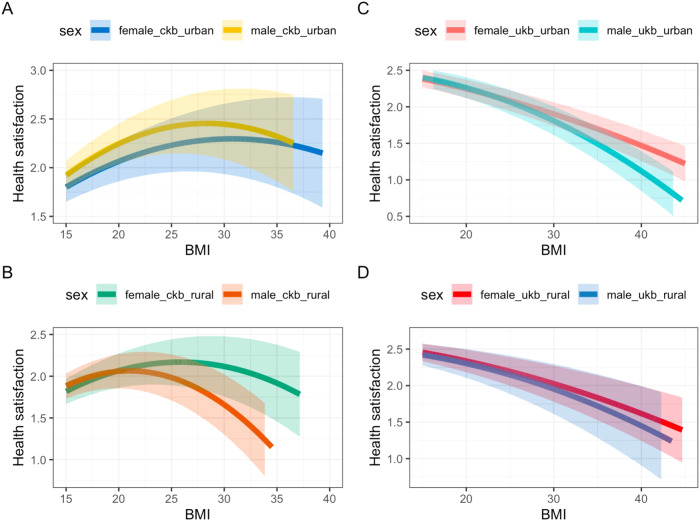
Non linear relationships between BMI and health satisfaction. **A** plot of the NLMR estimates using a control function approach in **A** Urban regions in the CKB, **B** Rural regions of the CKB, **C** Urban regions of the UKB and **D** Rural regions of the UKB, stratified by sex.

Non-linear MR provided some evidence for a non-linear relationship between BMI and health satisfaction in all individuals from both the CKB and UKB (Fig. 3 and Supplementary Table 12). In the CKB, we saw evidence of an inverted U-shaped relationship (*P* < 1.00E-15) whereas in the UKB, the relationship was mildly curvilinear but with a consistent inverse association across the full BMI range (*P* < 1.00E-15). Results were consistent when stratifying by sex and urban vs rural dwelling in the CKB and UKB (Fig. 3 and Supplementary Table 12). We found a significant difference in the non-linear relationships between BMI and health satisfaction observed in people of EAS ancestry and EUR ancestry across all regions and when stratifying by sex (Fig. 3 and Supplementary Table 12).

**Fig. 4:**
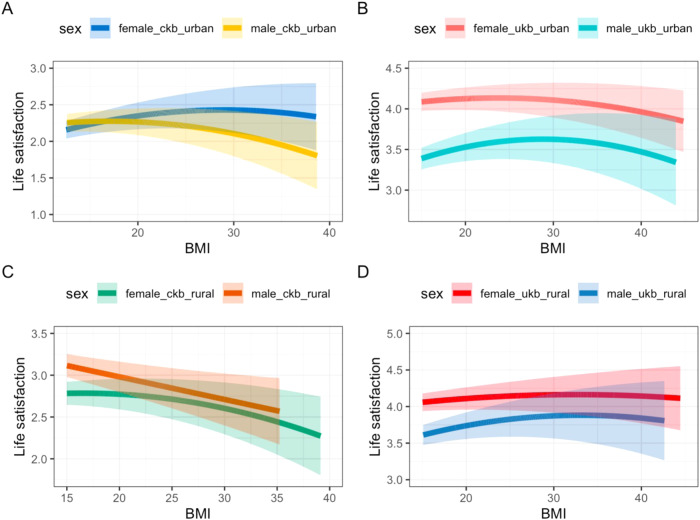
Non linear relationships between BMI and life satisfaction. A plot of the NLMR estimates using a CF approach of BMI to life satisfaction in **A** Urban regions in the CKB, **B** Rural regions of the CKB, **C** Urban regions of the UKB, and **D** Rural regions of the UKB, stratified by sex.

Similarly, we found some evidence for non-linear relationships between BMI and life satisfaction, in all individuals from both the CKB (*P* = 1.00E-03) and UKB (*P* < 1.00E-15; Fig. 4 and Supplementary Table 13). The estimate in the CKB was driven by females (*P* = 5.73E-06) with no evidence for a non-linear relationship in males (*P* = 0.81). The results were consistent when stratifying by sex and home location (Fig. 4 and Supplementary Table 13). There was a significant difference between the CKB and UKB non-linear estimates for BMI to life satisfaction (Fig. 4 and Supplementary Table 13). Further, although the *p* values for the quadratic estimates were significant, the confidence intervals were wide (Fig. 4).

## Discussion

This study tested the causal role of higher BMI on subjective well-being outcomes in two large cohorts of people of East Asian and European ancestries. We have provided evidence that (a) it is important to consider setting specific causality where the relationship may not be purely biological and (b) that where possible it is important to consider non-linear relationships. In particular, null estimates in linear models may mask a combination of strong positive and negative effects at different BMIs.

The pathways through which BMI could influence life satisfaction are complex but include weight-related stigma and social norms. Health satisfaction may be influenced by the direct health consequences of higher body weight, but an association between higher BMI and lower health satisfaction could also partially reflect social norms and the messages about health-related consequences of obesity received from public health campaigns and other sources. Given that stigma, social norms and public health messages are likely to differ according to context, we may expect these relationships to be specific to a setting. Further, the perceived impact of increasing weight may vary according to whether someone is currently under or overweight [38–40]. Hence, it is important to investigate these relationships in diverse ancestries as the BMI to well-being relationship may not be purely biological and could be subject to setting-specific causality. To our knowledge, this is the first-time causal inference methods have been used in people of East Asian ancestry to tease apart the role of BMI in well-being. Here, using one-sample Mendelian randomisation methods in up to 100,574 individuals in the CKB, we provide tentative evidence that higher adiposity increases health satisfaction in people of East Asian descent, especially in females. By contrast, higher BMI was associated with lower life satisfaction in people of East Asian ancestry, especially in males. This contrasts with people of European ancestry, where higher BMI leads to lower health satisfaction, with no association between BMI and life satisfaction. In both studies we provide evidence of non-linear relationships between BMI and health and life satisfaction, suggesting the importance of considering both high and low BMI in relation to well-being.

The association between higher BMI and higher health satisfaction in people of East Asian ancestry fits with some previous observational literature [18, 41]. Whilst in high-income countries the “thin ideal” has been a cultural symbol [42], in low- and middle-income countries higher BMI may be considered a sign of wealth and better health, and hence weight-stigma may be less common. China’s history of famine and scarcity of food has led to the common perception that “happiness makes you fat” [43–45], which may also imply that being fatter makes you happy. This study suggests that currently within many regions of China higher BMI is contributing to higher health satisfaction and therefore, the public health response to the growing obesity problem in China is likely to need to differ from higher income countries such as the UK, where we see an inverse association between BMI and health satisfaction. However, this is very complex, with differing relationships within different regions of China. Furthermore, the social patterning of body weight changes over the course of economic development; as a country’s wealth increases, obesity moves from being more common in higher socioeconomic groups to being associated with socioeconomic disadvantage [46]. This suggests that as China develops as an economy we may see a directional switch in the association, so whilst this study demonstrates differences in effect sizes between people of East Asian and European ancestry now, this may change in the future.

The pathways from adiposity to subjective well-being could be explained by obesity-related problems such as diabetes, musculoskeletal problems, etc. or social factors such as weight-related stigma. We have previously used genetic variants that are associated with higher adiposity but a favourable metabolic profile, and these remained associated with poorer well-being [8]. The physical health consequences of higher BMI have been shown to be similar across the UK and China [47, 48]. Thus, our results suggest that the association of higher BMI with lower health satisfaction in the UK is not purely due to the physical health consequences of obesity driving the association in Europeans, with potential societal influences, perceptions and stigma hypothesised to contribute to the relationship between higher BMI and poorer well-being. The different direction of effects in China suggests that these processes of weight stigma and its consequences differ across the UK and China.

Our study further highlights the contrast in findings between EAS and EUR ancestry populations where higher BMI is associated with lower life satisfaction in the CKB but, in the UKB we saw no association between BMI and life satisfaction. Again, we provide evidence for sex- and region-specific relationships in the CKB with higher BMI associating with lower life satisfaction in males only. The male estimate was driven by males living in rural regions of China. However, when removing Hunan, the rural male estimate was attenuated to the null. A previous study concluded that low-income people are less inclined to be overweight in China and tend to have a healthier diet as income constraints lead them to consume food with lower calories and nutrition [49]. Furthermore, low incomes limit excess food consumption and increase physically demanding labour whereas high incomes increase access to food and allow avoidance of physically demanding labour [50]. However, there was a low prevalence of people in the region of Hunan reporting to be unsatisfied (0.8%) and this may bias our estimate. Further work is required to confirm these findings.

This study also highlights the importance of considering non-linear models in causal inference. Here, we found some evidence that the relationship between BMI and (a) health and (b) life satisfaction was non-linear with both high and low BMI associating with lower health/life satisfaction in individuals of East Asian and European ancestry. Further, we demonstrated differences in the non-linear relationship between BMI and health/life satisfaction in individuals of East Asian and European descent, with an inverted U-shaped relationship in individuals of East Asian ancestry, whilst in Europeans the relationship was mildly curvilinear but with a consistent inverse association across the full BMI range. In Europeans, this relationship may be explained by the thin-ideal internalisation [41, 51] hypothesis, according to which individuals in Western countries, particularly women, cognitively “buy into” socially defined ideals of attractiveness, i.e., being thin. This could explain why individuals of low BMI in the UKB report higher health satisfaction than individuals with low BMI in the CKB. However, this difference may also be explained by the responses being scaled differently or the questions being perceived/understood differently in people of East Asian and European ancestry. Whilst in high-income countries the “thin ideal” has been a cultural symbol, in low- and middle-income countries people do not have such a negative attitude towards having a higher BMI as it may be considered a sign of wealth and better health [43, 52, 53]. Furthermore, observational non-linear analyses suggested that the relationship between BMI and well-being outcomes may be more complex with fractional polynomial models, in some sub-analyses, better explaining the relationship than quadratic models in both the CKB and UKB. Also, although the quadratic term was significant in many of the NLMR analyses the confidence intervals were wide and indicated uncertainty in our analyses. Future work should aim to explore more complex models such as polynomials in a causal inference setting.

This study benefits from individual level data in both the CKB and UKB which enabled sex-specific analyses, non-linear analyses, and further regional analyses, to allow us to comprehensively test the role of higher BMI on well-being outcomes. We acknowledge some limitations in our work. The question used to define our measure of life satisfaction differed in the CKB and UKB, such that we cannot be sure any differences in the relationship between BMI and life satisfaction in people of East Asian and European ancestry populations are not due to our definitions not accurately capturing life satisfaction in both cohorts. Further, in the CKB there were a low percentage of individuals reporting to be “very unsatisfied” (0.29%), especially when analysing certain regions and therefore the power of our sex-specific regional analyses for life satisfaction is limited, and the by region analyses are exploratory, purely to try to understand the main results. Using 1-sample MR methods in the CKB and UKB analyses we were unable to account for potential pleiotropy in our models. However, previous work in the UKB using more pleiotropy robust methods provided consistent estimates with higher BMI associating with lower health satisfaction. Furthermore, we recognise that one-sample MR estimates of BMI to life satisfaction in people of EAS ancestry were in the opposite direction to the observational estimates within CKB. This may be a result of (a) unmeasured or residual confounding within our observational analyses [54] or (b) reverse causation. Accurately accounting for this in our observational analyses was not possible, although adjustment for socioeconomic factors did attenuate the observational estimates towards the null, tending to support the unmeasured confounding hypothesis. Whilst we considered all four key assumptions of MR, we were only able to use available data to test the exclusion restriction assumption and it is possible that the BMI GRS may associate with unmeasured confounders. Additionally, genetic associations can be confounded by demographic and family-level processes [55], for example indirect effects of parents’ genotype on offspring phenotype via environmental pathways which may violate the independence assumption specifically. Circumventing these sources of bias requires genotype data on multiple members of the same family, which was not available here. We acknowledge that here, we cannot exclude reverse causal pathways as we were unable to test the role of well-being on BMI using genetic approaches. Due to the large number of highly related analyses, it would be challenging to determine an exact threshold for multiple testing correction. However, we recognise the importance of this issue and future work should further investigate these findings with a focus on accounting for multiple testing. Furthermore, while our study makes robust use of the available data, it is important to note that the choice of 12 PCs, though justified, may not fully capture all aspects of genetic variation in the CKB dataset, given the vast size and complex ancestral diversity of the Chinese population. Although our analysis of health satisfaction is based on the subset of UKB participants who completed the MHQ, we believe that the sample size is still large enough to detect general trends. However, we must note that these findings may not be representative of the broader UKB population due to potential participation biases [56]. Finally, the variants included in the BMI GRS, used in CKB, were identified in a European Genome Wide Association Study (GWAS) of BMI and therefore may not accurately represent associations in individuals of East Asian ancestry. However, these were weighted by and aligned to the trait raising allele in the CKB, meaning that the BMI GRS was strongly associated with BMI in CKB (F-stat>100). Future work should aim to use an external East Asian cohort to discover instruments for BMI.

Overall, our study suggests potential setting-specific causality in the relationship between body weight and subjective well-being, with robust differences observed between East Asians and Europeans when considering very similar outcomes. This study highlights the importance of testing causal relationships in different ancestries, as casual nature of relationships, especially relationships influenced by social processes may be setting-specific. We also provide evidence for considering non-linear relationships in MR studies, with this study highlighting the importance of both high and low BMI for well-being in both people of East Asian and European ancestry.

## Supplementary information

Supplementary methods

Supplementary tables and figures

## Data Availability

The data that support the findings of this study are available from the China Kadoorie Biobank Collaborative Group but restrictions apply to the availability of these data, which were used under license for the current study, and so are not publicly available. Summary data are however available from the authors upon reasonable request and with permission of the China Kadoorie Biobank Collaborative Group.
