# Supplementary material for: BMI and well-being in people of East Asian and European ancestry: a Mendelian randomisation study
Source: Transl Psychiatry. 2023 Jul 11;13:251. doi: 10.1038/s41398-023-02539-7 (PMC10336095; doi:10.1038/s41398-023-02539-7)
Supplement: Supplementary file 1 — Supplementary methods [file 41398_2023_2539_MOESM1_ESM.docx]

BMI and Well-being in people of East Asian and European Ancestry: A Mendelian Randomisation Study

Jessica O’Loughlin^1^, Francesco Casanova^1^, Amanda Hughes^2^, Zammy Fairhurst-Hunter^3^, Liming Li^4^, Zhengming Chen^3,5^, China Kadoorie Biobank Collaborative Group, Jack Bowden^1^, Ed Watkins^6^, Rachel M. Freathy^1^, Laura D Howe^2^, Robin G Walters^3^, and Jessica Tyrrell^1^

^1^College of Biomedical and Clinical Sciences, Faculty of Health and Life Sciences, University of Exeter, United Kingdom

^2^MRC Integrative Epidemiology Unit (IEU), Population Health Sciences, Bristol Medical School, University of Bristol, United Kingdom

^3^Clinical Trial Service Unit and Epidemiological Studies Unit (CTSU), Nuffield Department of Population Health, University of Oxford, United Kingdom

^4^Department of Epidemiology and Biostatistics, School of Public Health, Peking University, Beijing

^5^MRC Population Health Research Unit, Nuffield Department of Population Health, University of Oxford, United Kingdom

^6^Department of Psychology, University of Exeter, United Kingdom

Corresponding Author: Dr Jessica Tyrrell (j.tyrrell@exeter.ac.uk)

Supplementary methods

Socioeconomic status (SES)

**China Kadoorie Biobank (CKB):**

Several measures of SES were considered in CKB. Firstly, we used a principal component factor analysis to condense data on six related variables, where participants were asked “Do you have a) health cover, b) your own home, c) a private toilet, d) your own phone, e) your own motor vehicle and f) have you had a recent holiday?”, with the option to respond yes (1) or no (0). Two eigenvalues were greater than 1. Here, we use factor one as a measure of SES with higher values representing higher SES. We also considered highest education where participants were asked “What is the highest level of school education you ever received?” with the options of “No formal school”, “Primary school”, “Middle school”, “High school”, “Technical school/college”, or “University”. We created a categorical variable 0 to 5 with 5 representing a higher level of education (University). Finally, we adjusted for a categorical household income variable (questionnaire-based) representing annual household income of <2,500 yuan, 2,500-4,999 yuan, 5,000-9,999 yuan, 10,000-19,999 yuan, 20,000-34,999 yuan and >=35,000 yuan.

Amongst genotyped participants, 100,574 had information on SES in CKB.

**UK Biobank (UKB):**

In the UKB SES was defined by the following:

- Townsend deprivation index (TDI) calculated immediately prior to participant joining UKB. Based on the preceding national census output areas. Each participant is assigned a score corresponding to the output area in which their postcode is located.
- Educational Attainment: Participants were asked “Which of the following qualifications do you have?” with the options to respond to one or more of the following options: “College or University degree”, “A levels/AS levels or equivalent”, “O levels/GCSEs or equivalent”, “CSEs or equivalent”, “NVQ or HND or HNC or equivalent”, “Other professional qualifications g: nursing, teaching”, “None of the above” and “Prefer not to answer”. We created a categorical variable 0-None, 1- national vocational qualification, 2-GCSE or A-levels and 3-degree/professional qualifications.
- Household Income: Participants were asked "What is the average total income before tax received by your household?" With the options to respond “less than £18,000”, “£18,000 to £29,999”, “£30,000 to £51,999”, “£52,000 to £100,000”, “greater than £100,000”, “don’t know” or “prefer not to answer”. We created a categorical variable (1 to 5) removing participants who responded “don’t know” or “prefer not to answer”

Amongst genotyped participants, 379,708 and 130,298 had information on SES and health satisfaction and life satisfaction in the UKB, respectively.

**Members of the China Kadoorie Biobank Collaborative Group**

**International Steering Committee:** Junshi Chen, Zhengming Chen (PI), Robert Clarke, Rory Collins, Yu Guo, Liming Li (PI), Chen Wang, Jun Lv, Richard Peto, Robin Walters.

**International Co-ordinating Centre, Oxford:** Daniel Avery, Maxim Barnard, Derrick Bennett, Ruth Boxall, Ka Hung Chan, Yiping Chen, Zhengming Chen, Johnathan Clarke; Robert Clarke, Huaidong Du, Ahmed Edris Mohamed, Hannah Fry, Simon Gilbert, Pek Kei Im, Andri Iona, Maria Kakkoura, Christiana Kartsonaki, Hubert Lam, Kuang Lin, James Liu, Mohsen Mazidi, Iona Millwood, Sam Morris, Qunhua Nie, Alfred Pozarickij, Paul Ryder, Saredo Said, Dan Schmidt, Becky Stevens, Iain Turnbull, Robin Walters, Baihan Wang, Lin Wang, Neil Wright, Ling Yang, Xiaoming Yang, Pang Yao.

**National Co-ordinating Centre, Beijing:** Xiao Han, Can Hou, Qingmei Xia, Chao Liu, Jun Lv, Pei Pei, Dianjianyi Sun, Canqing Yu.

**Regional Co-ordinating Centres:**

**Gansu:** Gansu Provincial CDC: Caixia Dong, Pengfei Ge, Xiaolan Ren. Maiji CDC: Zhongxiao Li, Enke Mao, Tao Wang, Hui Zhang, Xi Zhang. **Haikou:** Hainan Provincial CDC: Jinyan Chen, Ximin Hu, Xiaohuan Wang. Meilan CDC: Zhendong Guo, Huimei Li, Yilei Li, Min Weng, Shukuan Wu. **Harbin:** Heilongjiang Provincial CDC: Shichun Yan, Mingyuan Zou, Xue Zhou. Nangang CDC: Ziyan Guo, Quan Kang, Yanjie Li, Bo Yu, Qinai Xu. **Henan:** Henan Provincial CDC: Liang Chang, Lei Fan, Shixian Feng, Ding Zhang, Gang Zhou. Huixian CDC: Yulian Gao, Tianyou He, Pan He, Chen Hu, Huarong Sun, Xukui Zhang. **Hunan:** Hunan Provincial CDC: Biyun Chen, Zhongxi Fu, Yuelong Huang, Huilin Liu, Qiaohua Xu, Li Yin. Liuyang CDC: Huajun Long, Xin Xu, Hao Zhang, Libo Zhang. **Liuzhou:** Guangxi Provincial CDC: Naying Chen, Duo Liu, Zhenzhu Tang. Liuzhou CDC: Ningyu Chen, Qilian Jiang, Jian Lan, Mingqiang Li, Yun Liu, Fanwen Meng, Jinhuai Meng, Rong Pan, Yulu Qin, Ping Wang, Sisi Wang, Liuping Wei, Liyuan Zhou. **Qingdao:** Qingdao CDC: Liang Cheng, Ranran Du, Ruqin Gao, Feifei Li, Shanpeng Li, Yongmei Liu, Feng Ning, Zengchang Pang, Xiaohui Sun, Xiaocao Tian, Shaojie Wang, Yaoming Zhai, Hua Zhang, Licang CDC: Wei Hou, Silu Lv, Junzheng Wang. **Sichuan:** Sichuan Provincial CDC: Xiaoyu Chang, Xiaofang Chen, Xianping Wu, Ningmei Zhang. Pengzhou CDC: Xiaofang Chen, Jianguo Li, Jiaqiu Liu, Guojin Luo, Qiang Sun, Xunfu Zhong. **Suzhou:** Jiangsu Provincial CDC: Jian Su, Ran Tao, Ming Wu, Jie Yang, Jinyi Zhou, Yonglin Zhou. Suzhou CDC: Yihe Hu, Yujie Hua, Jianrong Jin, Fang Liu, Jingchao Liu, Yan Lu, Liangcai Ma, Aiyu Tang, Jun Zhang. **Zhejiang**: Zhejiang Provincial CDC: Weiwei Gong, Ruying Hu, Hao Wang, Meng Wang, Min Yu. Tongxiang CDC: Lingli Chen, Qijun Gu, Dongxia Pan, Chunmei Wang, Kaixu Xie, Xiaoyi Zhang.
