# Supplementary material for: BMI and well-being in people of East Asian and European ancestry: a Mendelian randomisation study
Source: Transl Psychiatry. 2023 Jul 11;13:251. doi: 10.1038/s41398-023-02539-7 (PMC10336095; doi:10.1038/s41398-023-02539-7)

BMI and Well-being in people of East Asian and European Ancestry: A Mendelian Randomisation Study

Jessica O’Loughlin^1^, Francesco Casanova^1^, Amanda Hughes^2^, Zammy Fairhurst-Hunter^3^, Liming Li^4^, Zhengming Chen^3,5^, China Kadoorie Biobank Collaborative Group, Jack Bowden^1^, Ed Watkins^6^, Rachel M. Freathy^1^, Laura D Howe^2^, Robin G Walters^3^, and Jessica Tyrrell^1^

^1^College of Biomedical and Clinical Sciences, Faculty of Health and Life Sciences, University of Exeter, United Kingdom

^2^MRC Integrative Epidemiology Unit (IEU), Population Health Sciences, Bristol Medical School, University of Bristol, United Kingdom

^3^Clinical Trial Service Unit and Epidemiological Studies Unit (CTSU), Nuffield Department of Population Health, University of Oxford, United Kingdom

^4^Department of Epidemiology and Biostatistics, School of Public Health, Peking University, Beijing

^5^MRC Population Health Research Unit, Nuffield Department of Population Health, University of Oxford, United Kingdom

^6^Department of Psychology, University of Exeter, United Kingdom

Corresponding Author: Dr Jessica Tyrrell (j.tyrrell@exeter.ac.uk)

Supplementary table 1: The BMI variants that reach P<1x10-8 in independent genome wide association studies in European ancestries that were extracted from the imputed CKB data.

| **chr** | **pos** | **SNP** | **Trait-raising allele** | **Other allele** |
| --- | --- | --- | --- | --- |
| 1 | 167280354 | rs10733051 | A | G |
| 1 | 197012111 | rs10754210 | G | A |
| 1 | 11284336 | rs10779751 | A | G |
| 1 | 225668524 | rs10915840 | A | G |
| 1 | 190239907 | rs10920678 | A | G |
| 1 | 119546842 | rs10923724 | C | T |
| 1 | 219633869 | rs11118308 | G | A |
| 1 | 65987164 | rs11208662 | C | G |
| 1 | 155983710 | rs11577179 | G | A |
| 1 | 101024370 | rs1158103 | G | A |
| 1 | 112318484 | rs12033257 | A | G |
| 1 | 107885018 | rs12035149 | G | C |
| 1 | 77557339 | rs12035349 | G | A |
| 1 | 173549827 | rs12039524 | A | G |
| 1 | 195047936 | rs12041258 | T | C |
| 1 | 74997762 | rs12042908 | A | G |
| 1 | 243533273 | rs12042959 | A | G |
| 1 | 1708801 | rs12044597 | G | A |
| 1 | 174478100 | rs12564992 | G | A |
| 1 | 118852975 | rs12731372 | T | C |
| 1 | 209543560 | rs17014375 | G | T |
| 1 | 107617707 | rs1730859 | G | A |
| 1 | 15966713 | rs17448682 | C | T |
| 1 | 107977075 | rs17531363 | A | C |
| 1 | 96943994 | rs1973993 | T | C |
| 1 | 97388226 | rs2030342 | T | C |
| 1 | 80791708 | rs2154297 | T | C |
| 1 | 46487552 | rs2275426 | A | G |
| 1 | 39569571 | rs2282231 | T | C |
| 1 | 62594677 | rs2481665 | C | T |
| 1 | 242986063 | rs2491864 | A | G |
| 1 | 72885281 | rs2590942 | T | G |
| 1 | 201841476 | rs2820311 | G | A |
| 1 | 82379446 | rs284227 | C | T |
| 1 | 45620134 | rs346722 | C | T |
| 1 | 243722892 | rs3753549 | C | T |
| 1 | 19510394 | rs3762396 | A | G |
| 1 | 110123971 | rs3768486 | G | A |
| 1 | 98320492 | rs4372296 | A | C |
| 1 | 33776728 | rs4653017 | T | C |
| 1 | 177889480 | rs543874 | G | A |
| 1 | 6715390 | rs6577584 | G | T |
| 1 | 151018861 | rs6587552 | A | G |
| 1 | 92976590 | rs6690764 | G | A |
| 1 | 47700027 | rs6700838 | C | T |
| 1 | 16828640 | rs696606 | A | G |
| 1 | 72837239 | rs7531118 | C | T |
| 1 | 49828663 | rs7531656 | A | G |
| 1 | 2444414 | rs7535528 | G | A |
| 1 | 8741401 | rs7556169 | G | A |
| 1 | 17301672 | rs761423 | T | C |
| 1 | 33307987 | rs785278 | T | A |
| 1 | 154991389 | rs905938 | C | T |
| 1 | 202116238 | rs9077 | G | A |
| 1 | 173473713 | rs912768 | C | G |
| 1 | 34602870 | rs9426003 | G | A |
| 1 | 23399932 | rs967605 | C | T |
| 2 | 51834839 | rs10168197 | G | C |
| 2 | 25150296 | rs10182181 | G | A |
| 2 | 61619267 | rs10190332 | G | T |
| 2 | 103496700 | rs10203277 | A | G |
| 2 | 229016917 | rs10211055 | C | T |
| 2 | 198585087 | rs10497807 | G | C |
| 2 | 6155557 | rs10929925 | C | A |
| 2 | 175000711 | rs10930641 | A | G |
| 2 | 220163543 | rs11889536 | A | G |
| 2 | 228891702 | rs12479233 | T | A |
| 2 | 102436738 | rs12615778 | A | G |
| 2 | 206084372 | rs12694021 | C | A |
| 2 | 61307982 | rs12713433 | T | C |
| 2 | 199169278 | rs12991989 | C | G |
| 2 | 632348 | rs13021737 | G | A |
| 2 | 62848319 | rs13417156 | C | T |
| 2 | 56603985 | rs13432055 | C | T |
| 2 | 81816251 | rs1371108 | A | C |
| 2 | 211983316 | rs1437929 | A | G |
| 2 | 105466005 | rs1451533 | A | G |
| 2 | 151198990 | rs16828086 | C | G |
| 2 | 46878616 | rs17035438 | G | A |
| 2 | 208255518 | rs17203016 | G | A |
| 2 | 35512183 | rs17327461 | C | T |
| 2 | 142293146 | rs17551974 | C | A |
| 2 | 50865334 | rs2194385 | A | C |
| 2 | 471514 | rs2724861 | G | A |
| 2 | 67825685 | rs2902021 | C | T |
| 2 | 5830599 | rs3922853 | C | A |
| 2 | 100830040 | rs4303732 | C | T |
| 2 | 28973883 | rs4372836 | T | C |
| 2 | 147907202 | rs453520 | C | T |
| 2 | 416815 | rs4639527 | G | A |
| 2 | 37046657 | rs4670626 | C | T |
| 2 | 58935282 | rs4671328 | T | G |
| 2 | 60164634 | rs4671358 | A | T |
| 2 | 211608379 | rs4673553 | G | T |
| 2 | 110010962 | rs4676084 | G | A |
| 2 | 603088 | rs4854326 | A | G |
| 2 | 59307725 | rs6545714 | A | G |
| 2 | 295255 | rs6548221 | A | G |
| 2 | 143960593 | rs6710871 | A | G |
| 2 | 40291940 | rs6713781 | C | G |
| 2 | 230663576 | rs6720868 | C | T |
| 2 | 172599615 | rs6738445 | T | C |
| 2 | 58481863 | rs6753170 | T | C |
| 2 | 512490 | rs7425440 | C | T |
| 2 | 86766153 | rs7557796 | T | C |
| 2 | 48954905 | rs7561278 | T | C |
| 2 | 204012790 | rs7564679 | G | A |
| 2 | 50735943 | rs7598402 | G | C |
| 2 | 213413231 | rs7599312 | G | A |
| 2 | 156018263 | rs7600699 | C | G |
| 2 | 55281901 | rs7601895 | C | G |
| 2 | 69562127 | rs7607351 | T | C |
| 2 | 219279097 | rs7607369 | A | G |
| 2 | 113955074 | rs902695 | G | A |
| 2 | 198166565 | rs919433 | A | G |
| 2 | 58792377 | rs929641 | A | G |
| 2 | 50233352 | rs930295 | A | C |
| 2 | 79482643 | rs934515 | A | G |
| 2 | 181607676 | rs9630985 | C | A |
| 2 | 51171962 | rs968972 | A | G |
| 2 | 207244783 | rs972540 | G | A |
| 2 | 60285100 | rs980329 | C | T |
| 2 | 188325474 | rs993954 | T | G |
| 3 | 135621417 | rs1014312 | G | C |
| 3 | 42308735 | rs10460960 | A | G |
| 3 | 6009092 | rs10510321 | T | C |
| 3 | 34700713 | rs10865858 | T | C |
| 3 | 134665159 | rs10935143 | A | G |
| 3 | 15873407 | rs11128760 | A | G |
| 3 | 21794904 | rs11711337 | A | T |
| 3 | 70539559 | rs11915371 | A | C |
| 3 | 56114861 | rs12488237 | C | T |
| 3 | 50080174 | rs12631248 | G | C |
| 3 | 49237334 | rs12637576 | C | T |
| 3 | 89331055 | rs12638746 | G | A |
| 3 | 193639623 | rs13072095 | T | C |
| 3 | 171129859 | rs13085472 | T | C |
| 3 | 51243837 | rs13095652 | C | T |
| 3 | 131758077 | rs1320903 | A | G |
| 3 | 85658230 | rs1375561 | T | C |
| 3 | 104606130 | rs1436343 | G | A |
| 3 | 62481063 | rs1452075 | T | C |
| 3 | 176341188 | rs1454148 | C | T |
| 3 | 94038085 | rs1454687 | C | G |
| 3 | 8138801 | rs1554193 | T | A |
| 3 | 136979939 | rs1600136 | C | A |
| 3 | 115071668 | rs16823670 | A | G |
| 3 | 141275436 | rs16851483 | T | G |
| 3 | 48732480 | rs17080319 | C | T |
| 3 | 136744386 | rs181732 | G | T |
| 3 | 12394840 | rs1899951 | T | C |
| 3 | 124687767 | rs1909586 | G | T |
| 3 | 123093541 | rs2124499 | G | C |
| 3 | 49936102 | rs2230590 | T | C |
| 3 | 49343175 | rs2230929 | G | A |
| 3 | 50420554 | rs2236950 | A | C |
| 3 | 184044433 | rs2293605 | C | T |
| 3 | 61236462 | rs2365389 | C | T |
| 3 | 183486117 | rs262956 | T | G |
| 3 | 53777176 | rs2680648 | T | C |
| 3 | 52815905 | rs2710323 | C | T |
| 3 | 42418446 | rs28350 | A | G |
| 3 | 154034950 | rs355777 | C | G |
| 3 | 115417865 | rs3772934 | T | C |
| 3 | 52084040 | rs3821841 | C | T |
| 3 | 81792112 | rs3849570 | C | A |
| 3 | 9514856 | rs3915844 | G | A |
| 3 | 173095123 | rs39654 | G | A |
| 3 | 108119071 | rs4273371 | C | T |
| 3 | 137215820 | rs4342060 | T | C |
| 3 | 119571541 | rs4624596 | T | C |
| 3 | 136907111 | rs4678297 | C | T |
| 3 | 20441050 | rs4858193 | C | T |
| 3 | 170744815 | rs5396 | C | T |
| 3 | 13345450 | rs580438 | T | C |
| 3 | 46742019 | rs6442021 | C | T |
| 3 | 48130893 | rs6442101 | C | T |
| 3 | 89442305 | rs6551410 | T | A |
| 3 | 196088464 | rs6764533 | A | G |
| 3 | 156893782 | rs6767619 | C | G |
| 3 | 28727523 | rs6786125 | G | C |
| 3 | 89443784 | rs6803870 | T | C |
| 3 | 116937546 | rs6804181 | A | T |
| 3 | 25106437 | rs6804842 | G | A |
| 3 | 156299313 | rs7615297 | G | C |
| 3 | 77624784 | rs775731 | C | T |
| 3 | 157985182 | rs827092 | T | C |
| 3 | 62713143 | rs925018 | G | C |
| 3 | 90428286 | rs9714342 | T | C |
| 3 | 41310470 | rs9814633 | A | G |
| 3 | 185834499 | rs9816226 | T | A |
| 3 | 51184893 | rs9827072 | A | G |
| 3 | 89392778 | rs9832305 | C | T |
| 3 | 50820486 | rs9838283 | A | G |
| 3 | 49088112 | rs9846123 | C | T |
| 3 | 136845666 | rs9881036 | G | A |
| 4 | 38692835 | rs1000096 | C | T |
| 4 | 45182527 | rs10938397 | G | A |
| 4 | 147376805 | rs11736228 | A | T |
| 4 | 65700865 | rs11945861 | G | A |
| 4 | 137083193 | rs1296328 | A | C |
| 4 | 162129844 | rs13110266 | A | G |
| 4 | 20263058 | rs1323068 | G | A |
| 4 | 30843533 | rs1345148 | C | T |
| 4 | 119101723 | rs1403846 | T | C |
| 4 | 145986668 | rs1455137 | C | A |
| 4 | 18514827 | rs1477887 | G | A |
| 4 | 55221467 | rs1492767 | C | T |
| 4 | 163038241 | rs17538472 | T | C |
| 4 | 44514468 | rs1866510 | C | T |
| 4 | 91253956 | rs1903579 | C | G |
| 4 | 55505360 | rs2192158 | G | A |
| 4 | 49064487 | rs2768950 | A | G |
| 4 | 102183594 | rs2850969 | T | C |
| 4 | 112713436 | rs326889 | C | T |
| 4 | 143663206 | rs331949 | T | C |
| 4 | 89054667 | rs4148155 | A | G |
| 4 | 6492739 | rs4524456 | G | A |
| 4 | 130731284 | rs4864201 | T | C |
| 4 | 28561990 | rs6448587 | C | A |
| 4 | 16600664 | rs6818414 | T | C |
| 4 | 153075491 | rs6827083 | G | A |
| 4 | 103936001 | rs6843738 | G | A |
| 4 | 52926216 | rs711347 | A | T |
| 4 | 102708997 | rs7377083 | A | C |
| 4 | 80794681 | rs7674623 | T | C |
| 4 | 180167906 | rs7683836 | G | A |
| 4 | 10491040 | rs7692088 | G | C |
| 4 | 115124089 | rs7694732 | A | G |
| 4 | 140881964 | rs769674 | T | A |
| 4 | 60253877 | rs925421 | A | G |
| 4 | 25428296 | rs9291467 | C | T |
| 5 | 153095918 | rs10035289 | A | G |
| 5 | 151254297 | rs10066835 | C | T |
| 5 | 168192944 | rs1014194 | A | C |
| 5 | 80818639 | rs12514473 | T | C |
| 5 | 88858208 | rs12655756 | A | T |
| 5 | 152274478 | rs12659802 | G | A |
| 5 | 136571959 | rs13163306 | G | A |
| 5 | 139080745 | rs13174863 | G | A |
| 5 | 87125578 | rs1423627 | C | T |
| 5 | 94206202 | rs159032 | T | C |
| 5 | 138061341 | rs160401 | C | T |
| 5 | 157516393 | rs1650586 | G | T |
| 5 | 158271680 | rs17056301 | C | T |
| 5 | 86908920 | rs17285919 | T | C |
| 5 | 95933162 | rs174415 | A | T |
| 5 | 167293652 | rs17525725 | G | A |
| 5 | 86420392 | rs17591778 | A | G |
| 5 | 86754835 | rs186543 | G | C |
| 5 | 144484261 | rs2190788 | T | G |
| 5 | 87988733 | rs2304607 | G | A |
| 5 | 75003678 | rs2307111 | T | C |
| 5 | 64168193 | rs2367112 | G | T |
| 5 | 167352783 | rs248139 | A | G |
| 5 | 141777439 | rs254428 | T | G |
| 5 | 66175682 | rs25832 | A | G |
| 5 | 95856501 | rs2611742 | C | T |
| 5 | 133865452 | rs329124 | A | G |
| 5 | 96080883 | rs3822683 | G | A |
| 5 | 140992235 | rs3844598 | A | G |
| 5 | 107439012 | rs40067 | G | A |
| 5 | 27185904 | rs4518345 | G | A |
| 5 | 138372345 | rs4643949 | T | C |
| 5 | 87932809 | rs4916661 | G | T |
| 5 | 95728898 | rs6235 | G | C |
| 5 | 60712212 | rs6449531 | G | A |
| 5 | 119372533 | rs6595205 | G | C |
| 5 | 130356413 | rs6877851 | G | C |
| 5 | 59208302 | rs6879326 | C | T |
| 5 | 63932234 | rs6888159 | C | G |
| 5 | 86727027 | rs7702514 | T | C |
| 5 | 106506697 | rs7710595 | A | C |
| 5 | 122733317 | rs7711753 | G | A |
| 5 | 153537893 | rs7715256 | G | T |
| 5 | 137631073 | rs7716275 | T | G |
| 5 | 165185571 | rs7727781 | T | C |
| 5 | 43191033 | rs7730004 | T | C |
| 5 | 170459675 | rs7730898 | A | G |
| 6 | 154309808 | rs10499276 | T | C |
| 6 | 20705590 | rs11753081 | T | G |
| 6 | 57642076 | rs12207241 | G | A |
| 6 | 34644749 | rs12215331 | T | C |
| 6 | 51822297 | rs1266922 | G | A |
| 6 | 137675541 | rs13201877 | G | A |
| 6 | 97374850 | rs13203153 | G | A |
| 6 | 97753223 | rs13209872 | G | C |
| 6 | 126089285 | rs13209968 | C | G |
| 6 | 93913200 | rs1324110 | G | C |
| 6 | 51825285 | rs1358808 | G | C |
| 6 | 35619554 | rs1475774 | G | A |
| 6 | 54724405 | rs1503139 | G | A |
| 6 | 104799007 | rs156151 | C | G |
| 6 | 27293049 | rs17739298 | C | G |
| 6 | 87606842 | rs1853639 | G | A |
| 6 | 27859568 | rs200968 | T | C |
| 6 | 40348653 | rs2033529 | G | A |
| 6 | 34975415 | rs2140418 | T | C |
| 6 | 153381622 | rs2185027 | C | A |
| 6 | 12124855 | rs2228213 | G | A |
| 6 | 50566383 | rs2295896 | A | G |
| 6 | 120213880 | rs2357760 | A | G |
| 6 | 51638877 | rs2397061 | T | C |
| 6 | 51160682 | rs2504674 | G | C |
| 6 | 50820940 | rs2635727 | C | T |
| 6 | 55169801 | rs2653365 | C | T |
| 6 | 131897278 | rs2781668 | T | C |
| 6 | 34617144 | rs2814992 | G | A |
| 6 | 19078274 | rs2842385 | G | A |
| 6 | 124925032 | rs2875762 | C | G |
| 6 | 28059458 | rs3734572 | C | T |
| 6 | 108996963 | rs3800229 | T | G |
| 6 | 35512955 | rs3807049 | C | T |
| 6 | 31322367 | rs3819299 | T | G |
| 6 | 33554147 | rs419261 | C | T |
| 6 | 53693410 | rs4278019 | A | T |
| 6 | 57964315 | rs4339513 | T | C |
| 6 | 160774459 | rs487060 | T | C |
| 6 | 58060143 | rs6459326 | C | G |
| 6 | 130349119 | rs6569648 | C | T |
| 6 | 23876240 | rs6900723 | T | C |
| 6 | 58705746 | rs6904676 | A | C |
| 6 | 27527205 | rs6908295 | A | C |
| 6 | 104493098 | rs6919443 | G | A |
| 6 | 73742334 | rs6921533 | T | C |
| 6 | 69968484 | rs6922214 | A | G |
| 6 | 51843542 | rs6922855 | G | A |
| 6 | 34683635 | rs6938239 | A | G |
| 6 | 27408181 | rs760880 | T | G |
| 6 | 51874745 | rs765332 | T | G |
| 6 | 41133806 | rs7748777 | A | G |
| 6 | 21919387 | rs7760082 | G | A |
| 6 | 53990465 | rs816364 | G | A |
| 6 | 35033854 | rs820077 | G | A |
| 6 | 40080069 | rs847747 | G | T |
| 6 | 28296863 | rs853679 | A | C |
| 6 | 98539519 | rs901630 | T | C |
| 6 | 58298846 | rs926279 | A | G |
| 6 | 83433228 | rs9294260 | G | A |
| 6 | 42676480 | rs9349239 | G | A |
| 6 | 90296588 | rs9362662 | A | G |
| 6 | 13189275 | rs9367368 | T | C |
| 6 | 51546123 | rs9370042 | G | C |
| 6 | 35672330 | rs9394312 | C | G |
| 6 | 9952059 | rs9396763 | C | A |
| 6 | 19211776 | rs9460306 | T | C |
| 6 | 9510030 | rs9463175 | C | T |
| 6 | 55013291 | rs9475173 | A | G |
| 6 | 73922654 | rs9688431 | T | C |
| 6 | 50803050 | rs987237 | G | A |
| 6 | 43757896 | rs998584 | C | A |
| 7 | 49616203 | rs10269783 | A | G |
| 7 | 1273845 | rs10275044 | T | A |
| 7 | 69598328 | rs1035010 | T | C |
| 7 | 100804430 | rs1048365 | T | C |
| 7 | 109173373 | rs10953620 | C | A |
| 7 | 103417557 | rs11496125 | T | C |
| 7 | 147668180 | rs11773362 | C | T |
| 7 | 26526960 | rs12666574 | G | A |
| 7 | 114349212 | rs12705987 | A | T |
| 7 | 74094721 | rs13227433 | G | T |
| 7 | 99064466 | rs13240600 | A | G |
| 7 | 93232057 | rs13247665 | C | T |
| 7 | 77417584 | rs1544459 | C | T |
| 7 | 131619847 | rs1593304 | G | A |
| 7 | 75101065 | rs17207196 | C | T |
| 7 | 138794149 | rs1814170 | A | T |
| 7 | 77829768 | rs1852006 | G | A |
| 7 | 121964349 | rs1899689 | T | C |
| 7 | 32368524 | rs215632 | G | A |
| 7 | 126721231 | rs2283093 | T | C |
| 7 | 35080931 | rs329277 | G | T |
| 7 | 137424509 | rs3800649 | A | G |
| 7 | 50564204 | rs3807566 | G | T |
| 7 | 21470536 | rs40245 | A | T |
| 7 | 94033031 | rs411717 | C | T |
| 7 | 24354300 | rs4307239 | G | A |
| 7 | 70040558 | rs4718966 | T | C |
| 7 | 3125220 | rs4722398 | T | C |
| 7 | 27231762 | rs4722672 | T | C |
| 7 | 150668514 | rs4725984 | C | T |
| 7 | 2103668 | rs6461115 | A | G |
| 7 | 5542513 | rs6463489 | T | C |
| 7 | 78144371 | rs6963840 | T | C |
| 7 | 17287106 | rs6968554 | G | A |
| 7 | 77055885 | rs740157 | A | G |
| 7 | 73058017 | rs7777102 | G | A |
| 7 | 6418275 | rs7784465 | C | T |
| 7 | 897847 | rs7796608 | A | G |
| 7 | 78121458 | rs7805441 | T | C |
| 7 | 44784697 | rs799449 | T | C |
| 7 | 28196413 | rs849135 | A | G |
| 7 | 131896813 | rs874454 | G | A |
| 7 | 130466854 | rs972283 | A | G |
| 7 | 71888157 | rs993931 | G | A |
| 8 | 14324437 | rs10110727 | G | A |
| 8 | 118946541 | rs10955841 | A | G |
| 8 | 10647823 | rs11250076 | A | G |
| 8 | 23389571 | rs11781222 | C | T |
| 8 | 118863061 | rs11781699 | C | T |
| 8 | 10788875 | rs11783247 | C | T |
| 8 | 15197115 | rs11987383 | A | C |
| 8 | 11617240 | rs12458 | A | T |
| 8 | 132879047 | rs12675063 | T | A |
| 8 | 95582606 | rs12680842 | A | G |
| 8 | 10626280 | rs12682565 | A | G |
| 8 | 14095900 | rs13263601 | A | C |
| 8 | 30856464 | rs1362910 | A | G |
| 8 | 9511654 | rs1394 | A | G |
| 8 | 9785503 | rs1399054 | A | G |
| 8 | 77228222 | rs1405348 | G | A |
| 8 | 73439070 | rs1431659 | A | G |
| 8 | 26334167 | rs1594830 | G | C |
| 8 | 4288577 | rs1658820 | T | G |
| 8 | 138215228 | rs16906845 | G | A |
| 8 | 81375457 | rs16907751 | C | T |
| 8 | 67202787 | rs16932761 | A | G |
| 8 | 89461609 | rs1700137 | C | T |
| 8 | 4137396 | rs17069831 | C | T |
| 8 | 27167942 | rs17446091 | T | C |
| 8 | 28118130 | rs2100814 | A | G |
| 8 | 11060638 | rs2409730 | C | A |
| 8 | 15536311 | rs2543132 | G | C |
| 8 | 85696337 | rs2634047 | G | C |
| 8 | 116662038 | rs2721965 | A | C |
| 8 | 101947453 | rs3134353 | T | A |
| 8 | 116559435 | rs3808434 | A | G |
| 8 | 144910239 | rs4076358 | A | G |
| 8 | 20639811 | rs4366093 | C | T |
| 8 | 64720693 | rs4737183 | G | A |
| 8 | 11828200 | rs4841659 | T | C |
| 8 | 9790041 | rs497417 | A | T |
| 8 | 87519542 | rs7006629 | T | C |
| 8 | 85077686 | rs733594 | T | C |
| 8 | 8380471 | rs7827182 | G | C |
| 8 | 34503776 | rs7844647 | T | C |
| 8 | 38332318 | rs881301 | C | T |
| 8 | 142630782 | rs903959 | A | T |
| 8 | 10864363 | rs9657542 | G | C |
| 9 | 103061366 | rs10118701 | G | A |
| 9 | 92191256 | rs10797115 | C | T |
| 9 | 126096522 | rs10818810 | G | A |
| 9 | 127049237 | rs10818938 | A | G |
| 9 | 81367391 | rs10867256 | C | T |
| 9 | 16719445 | rs10962549 | T | C |
| 9 | 104396304 | rs10989568 | A | G |
| 9 | 14651283 | rs11790280 | C | T |
| 9 | 140646121 | rs11792069 | G | A |
| 9 | 37183628 | rs13290794 | G | A |
| 9 | 129467340 | rs13292976 | T | C |
| 9 | 28410996 | rs1412235 | C | G |
| 9 | 83263089 | rs17351791 | A | C |
| 9 | 8845911 | rs1865341 | T | C |
| 9 | 120378483 | rs1928295 | T | C |
| 9 | 73791849 | rs2174307 | C | G |
| 9 | 129940416 | rs3739555 | T | G |
| 9 | 88897891 | rs3739733 | A | G |
| 9 | 129390800 | rs3829849 | T | C |
| 9 | 129419025 | rs3902840 | A | G |
| 9 | 101481205 | rs450231 | G | A |
| 9 | 128616073 | rs4515655 | T | C |
| 9 | 133783566 | rs4740383 | A | G |
| 9 | 96482633 | rs4744275 | A | G |
| 9 | 27612918 | rs483752 | T | C |
| 9 | 15670492 | rs6474945 | G | T |
| 9 | 111932342 | rs6477694 | C | T |
| 9 | 109072075 | rs7024334 | T | G |
| 9 | 6959840 | rs7042372 | A | G |
| 9 | 122631560 | rs7865157 | C | T |
| 9 | 94180627 | rs7869771 | A | C |
| 9 | 131027982 | rs7871866 | C | G |
| 9 | 27777012 | rs7874154 | C | T |
| 10 | 102635475 | rs10883553 | A | C |
| 10 | 104412049 | rs10883759 | G | A |
| 10 | 118672531 | rs10886017 | A | C |
| 10 | 88096047 | rs10887578 | C | G |
| 10 | 102452136 | rs11190661 | T | C |
| 10 | 2585792 | rs11251352 | A | G |
| 10 | 76047464 | rs12098284 | C | T |
| 10 | 104685299 | rs12411886 | A | C |
| 10 | 19776828 | rs12776880 | A | T |
| 10 | 102395440 | rs17094222 | C | T |
| 10 | 105033015 | rs1712517 | C | T |
| 10 | 126594078 | rs17636031 | C | T |
| 10 | 53680085 | rs1937684 | T | A |
| 10 | 100017453 | rs1983864 | T | G |
| 10 | 65314711 | rs2163188 | C | G |
| 10 | 93032943 | rs2631681 | C | T |
| 10 | 27318776 | rs3781099 | T | C |
| 10 | 33862727 | rs3851083 | G | A |
| 10 | 99769388 | rs577525 | T | C |
| 10 | 103984060 | rs7083450 | T | C |
| 10 | 21821274 | rs7084454 | A | G |
| 10 | 16750129 | rs7893571 | G | T |
| 10 | 114758349 | rs7903146 | C | T |
| 10 | 99032375 | rs793520 | A | G |
| 10 | 125220036 | rs845084 | A | G |
| 10 | 105675946 | rs9419958 | T | C |
| 10 | 103206115 | rs9787495 | G | A |
| 11 | 118913993 | rs1003081 | C | T |
| 11 | 11796727 | rs1037587 | T | C |
| 11 | 55091574 | rs10459012 | C | A |
| 11 | 115044850 | rs1048932 | C | A |
| 11 | 45706453 | rs10769165 | T | C |
| 11 | 89966202 | rs10830452 | G | A |
| 11 | 17394073 | rs10832778 | G | C |
| 11 | 43551416 | rs10838122 | C | T |
| 11 | 48286256 | rs10838852 | C | T |
| 11 | 49994823 | rs10839472 | T | C |
| 11 | 2234690 | rs10840606 | G | A |
| 11 | 28629115 | rs11030385 | G | A |
| 11 | 46895378 | rs11039014 | G | A |
| 11 | 64082807 | rs11600990 | C | T |
| 11 | 134601012 | rs12364470 | G | T |
| 11 | 115623272 | rs12417072 | A | G |
| 11 | 117017530 | rs12420725 | G | A |
| 11 | 46422686 | rs12574668 | C | A |
| 11 | 8694073 | rs12575252 | G | C |
| 11 | 43728534 | rs12577642 | A | T |
| 11 | 86133416 | rs1452134 | T | C |
| 11 | 48901553 | rs1473579 | A | G |
| 11 | 29158495 | rs1552717 | A | T |
| 11 | 131957293 | rs1625427 | T | C |
| 11 | 46520302 | rs17197116 | C | T |
| 11 | 30243868 | rs1782507 | G | T |
| 11 | 69481969 | rs1789165 | A | G |
| 11 | 56973793 | rs1943477 | C | T |
| 11 | 32131303 | rs223051 | T | C |
| 11 | 70563286 | rs2440885 | A | G |
| 11 | 93221105 | rs2605603 | G | A |
| 11 | 122765667 | rs3134438 | A | C |
| 11 | 133827733 | rs3802924 | C | A |
| 11 | 56446833 | rs4542429 | C | T |
| 11 | 132641959 | rs4936175 | C | T |
| 11 | 121942512 | rs4936671 | C | G |
| 11 | 56206141 | rs4939051 | C | G |
| 11 | 65886662 | rs524281 | C | A |
| 11 | 55323307 | rs551137 | T | C |
| 11 | 117267884 | rs573455 | G | A |
| 11 | 69299771 | rs587230 | A | G |
| 11 | 27679916 | rs6265 | C | T |
| 11 | 65594820 | rs7102454 | C | T |
| 11 | 78040259 | rs7117238 | G | A |
| 11 | 49459474 | rs7120873 | C | T |
| 11 | 66662731 | rs7122539 | A | G |
| 11 | 72444583 | rs7123876 | C | T |
| 11 | 27677041 | rs7124442 | C | T |
| 11 | 47529947 | rs7124681 | A | C |
| 11 | 47836302 | rs7131262 | T | A |
| 11 | 113234679 | rs719802 | T | C |
| 11 | 48630323 | rs7478904 | C | T |
| 11 | 49620595 | rs7924371 | C | T |
| 11 | 122522375 | rs7941030 | C | T |
| 11 | 28763321 | rs7948120 | C | T |
| 11 | 13294268 | rs900144 | T | C |
| 12 | 17212881 | rs10744146 | G | A |
| 12 | 97586257 | rs10745785 | C | T |
| 12 | 33379440 | rs10772055 | G | C |
| 12 | 124506631 | rs10773049 | C | T |
| 12 | 17360254 | rs10840674 | G | A |
| 12 | 24060075 | rs10842240 | C | G |
| 12 | 112771063 | rs10850031 | G | T |
| 12 | 39428802 | rs10876418 | C | T |
| 12 | 69642315 | rs10878946 | T | C |
| 12 | 82465797 | rs11115176 | T | C |
| 12 | 39982413 | rs11172702 | G | A |
| 12 | 939480 | rs11611246 | T | G |
| 12 | 38242029 | rs12306932 | C | T |
| 12 | 112019799 | rs12369009 | T | G |
| 12 | 14413931 | rs12422552 | C | G |
| 12 | 111780998 | rs1558236 | C | G |
| 12 | 38529333 | rs17096549 | A | G |
| 12 | 110046698 | rs17608150 | C | T |
| 12 | 68205604 | rs1819844 | A | G |
| 12 | 48180508 | rs2240108 | T | C |
| 12 | 2152655 | rs2429150 | C | A |
| 12 | 23067302 | rs2467110 | T | C |
| 12 | 90595383 | rs2731222 | A | C |
| 12 | 41880909 | rs2733287 | C | A |
| 12 | 111800258 | rs3809272 | A | G |
| 12 | 51593616 | rs4077093 | T | G |
| 12 | 54653258 | rs4759073 | G | A |
| 12 | 56508409 | rs4759228 | G | C |
| 12 | 103658096 | rs4764949 | A | G |
| 12 | 114437708 | rs4766710 | A | G |
| 12 | 18789007 | rs621042 | A | C |
| 12 | 111768973 | rs6490055 | A | G |
| 12 | 99560183 | rs651548 | A | G |
| 12 | 39159190 | rs6580755 | C | T |
| 12 | 124409502 | rs7133378 | A | G |
| 12 | 20473758 | rs7134375 | A | C |
| 12 | 50247468 | rs7138803 | A | G |
| 12 | 89899912 | rs7313924 | C | G |
| 12 | 39329294 | rs7958206 | A | G |
| 12 | 49987929 | rs7965658 | G | A |
| 12 | 133481917 | rs7968230 | G | A |
| 12 | 118409640 | rs7973955 | G | A |
| 12 | 60964108 | rs7975187 | A | G |
| 12 | 117579274 | rs884282 | T | C |
| 13 | 28047269 | rs1006353 | A | G |
| 13 | 31033232 | rs1045411 | C | T |
| 13 | 54694130 | rs10467530 | C | G |
| 13 | 28011963 | rs1218822 | A | G |
| 13 | 54102206 | rs12429545 | A | G |
| 13 | 59425111 | rs1333423 | A | T |
| 13 | 78371890 | rs1668633 | T | C |
| 13 | 96922191 | rs1927790 | C | T |
| 13 | 111984244 | rs2479958 | G | A |
| 13 | 76386075 | rs629443 | T | G |
| 13 | 99120484 | rs7334078 | T | C |
| 13 | 65884191 | rs892261 | C | T |
| 13 | 28620036 | rs9507983 | C | T |
| 13 | 58402479 | rs9527706 | A | G |
| 13 | 79563749 | rs9530843 | C | A |
| 13 | 59178258 | rs9538141 | A | G |
| 13 | 66205704 | rs9540493 | A | G |
| 13 | 36230485 | rs9544915 | T | C |
| 13 | 28681228 | rs9554263 | G | C |
| 13 | 67472713 | rs9571687 | C | A |
| 13 | 33184288 | rs9595908 | T | C |
| 13 | 40783323 | rs9603697 | T | C |
| 14 | 97258752 | rs10131890 | C | A |
| 14 | 25928179 | rs10132280 | C | A |
| 14 | 79499850 | rs10146527 | T | C |
| 14 | 65910844 | rs11844682 | G | C |
| 14 | 72269668 | rs1205106 | A | G |
| 14 | 47272423 | rs12587412 | T | G |
| 14 | 29736838 | rs12885454 | C | A |
| 14 | 77529783 | rs17105272 | T | C |
| 14 | 33302882 | rs17522122 | T | G |
| 14 | 91512339 | rs1951455 | C | T |
| 14 | 40101060 | rs1956151 | A | G |
| 14 | 79903993 | rs2003616 | T | G |
| 14 | 103862322 | rs2010281 | G | A |
| 14 | 92428410 | rs2160077 | A | G |
| 14 | 62360464 | rs217671 | G | A |
| 14 | 65426216 | rs2412107 | G | T |
| 14 | 103246470 | rs3803286 | A | G |
| 14 | 99671788 | rs3850422 | G | A |
| 14 | 69789755 | rs3902951 | T | G |
| 14 | 29680331 | rs4981693 | A | G |
| 14 | 101539384 | rs7147503 | C | T |
| 14 | 82684748 | rs799132 | T | A |
| 15 | 77156899 | rs10519151 | A | T |
| 15 | 84580156 | rs11259933 | G | A |
| 15 | 66741387 | rs11629783 | C | G |
| 15 | 78012618 | rs11855853 | C | T |
| 15 | 92570921 | rs12101393 | C | G |
| 15 | 77254544 | rs12148386 | T | C |
| 15 | 46584787 | rs12439798 | T | G |
| 15 | 81058652 | rs12593036 | G | A |
| 15 | 62316035 | rs12595158 | C | T |
| 15 | 79432359 | rs12595749 | A | G |
| 15 | 68104367 | rs13329567 | C | T |
| 15 | 89928189 | rs150353 | G | T |
| 15 | 57120989 | rs1657930 | G | A |
| 15 | 68103632 | rs16951319 | T | C |
| 15 | 62150364 | rs17238110 | G | A |
| 15 | 76863838 | rs2459359 | G | C |
| 15 | 76150965 | rs2593280 | A | G |
| 15 | 41751678 | rs316611 | T | C |
| 15 | 60908307 | rs340025 | C | T |
| 15 | 51748610 | rs3736485 | A | G |
| 15 | 76755506 | rs403656 | A | G |
| 15 | 31843528 | rs4284600 | T | C |
| 15 | 77207277 | rs4886506 | T | G |
| 15 | 77799657 | rs4886869 | G | A |
| 15 | 27040082 | rs4906908 | G | T |
| 15 | 64849904 | rs6494481 | G | T |
| 15 | 78117685 | rs6495252 | C | T |
| 15 | 73093991 | rs7164727 | T | C |
| 15 | 95271404 | rs7181498 | T | C |
| 15 | 77915282 | rs8024932 | T | G |
| 15 | 61445514 | rs8033510 | T | C |
| 15 | 36402716 | rs8036040 | A | C |
| 16 | 6701400 | rs10083803 | C | T |
| 16 | 69174141 | rs10500548 | T | C |
| 16 | 29833714 | rs1057452 | A | G |
| 16 | 20255123 | rs11074446 | T | C |
| 16 | 62803841 | rs11075489 | C | T |
| 16 | 53805344 | rs11075986 | C | G |
| 16 | 71899586 | rs11642001 | A | G |
| 16 | 387867 | rs11866815 | C | T |
| 16 | 52548037 | rs12443621 | G | A |
| 16 | 3599655 | rs12448257 | A | G |
| 16 | 56489343 | rs12448738 | C | A |
| 16 | 67420603 | rs12920590 | T | C |
| 16 | 82438337 | rs12922346 | C | G |
| 16 | 31141993 | rs1549293 | C | T |
| 16 | 50986308 | rs1564981 | A | G |
| 16 | 53770578 | rs16952479 | T | A |
| 16 | 51926509 | rs17795934 | C | T |
| 16 | 24803620 | rs1862451 | G | A |
| 16 | 54153099 | rs2075205 | A | T |
| 16 | 49062590 | rs2080454 | C | A |
| 16 | 68381978 | rs2307022 | A | G |
| 16 | 2097158 | rs2516739 | G | A |
| 16 | 6509009 | rs2534760 | T | A |
| 16 | 68295598 | rs2863981 | G | A |
| 16 | 53818708 | rs3751813 | G | T |
| 16 | 3730613 | rs3794702 | T | A |
| 16 | 29994922 | rs3814883 | T | C |
| 16 | 82650384 | rs4783241 | G | C |
| 16 | 15129459 | rs4985155 | A | G |
| 16 | 76779612 | rs6564360 | G | A |
| 16 | 28857645 | rs7187776 | G | A |
| 16 | 24578458 | rs7195386 | T | C |
| 16 | 67316600 | rs7200919 | A | G |
| 16 | 82872628 | rs7206608 | G | C |
| 16 | 72996162 | rs756717 | G | A |
| 16 | 70514828 | rs7919 | A | C |
| 16 | 80752293 | rs8046061 | C | T |
| 16 | 53798523 | rs8047395 | A | G |
| 16 | 73606563 | rs825680 | A | T |
| 16 | 20050466 | rs868554 | G | C |
| 16 | 4015729 | rs879620 | T | C |
| 16 | 69556715 | rs889398 | C | T |
| 16 | 54234492 | rs907011 | T | G |
| 16 | 71965915 | rs952159 | A | G |
| 16 | 9724750 | rs977540 | G | A |
| 16 | 53831146 | rs9922708 | T | C |
| 16 | 23833071 | rs9927848 | C | A |
| 16 | 53825238 | rs9931164 | A | G |
| 16 | 20375351 | rs9931967 | T | G |
| 17 | 5283252 | rs1000940 | A | G |
| 17 | 28074563 | rs1038088 | G | T |
| 17 | 79202329 | rs1048775 | C | G |
| 17 | 51923847 | rs10515050 | C | T |
| 17 | 15943910 | rs1075901 | C | T |
| 17 | 34942595 | rs1106908 | G | A |
| 17 | 47090785 | rs11079849 | C | T |
| 17 | 31747629 | rs12453418 | G | A |
| 17 | 65870073 | rs12602912 | T | C |
| 17 | 77796889 | rs1285245 | G | C |
| 17 | 78611724 | rs12939549 | A | G |
| 17 | 39573713 | rs16966801 | A | G |
| 17 | 46252346 | rs208015 | T | C |
| 17 | 65694355 | rs2537847 | G | A |
| 17 | 71754545 | rs2619976 | T | C |
| 17 | 1846831 | rs4516268 | C | A |
| 17 | 35057883 | rs4796243 | G | A |
| 17 | 78640510 | rs4889782 | C | T |
| 17 | 79081724 | rs4969387 | G | C |
| 17 | 21261560 | rs4986044 | C | T |
| 17 | 73759552 | rs7209235 | A | G |
| 17 | 31460899 | rs7211567 | C | T |
| 17 | 2136065 | rs7217226 | G | T |
| 17 | 59497277 | rs757608 | G | A |
| 17 | 29349688 | rs8067737 | T | C |
| 17 | 42935059 | rs8069296 | T | C |
| 17 | 38160754 | rs8070454 | C | T |
| 17 | 55336155 | rs8071182 | A | G |
| 17 | 61728881 | rs8075273 | C | A |
| 17 | 5412361 | rs8079034 | T | C |
| 17 | 46051911 | rs886444 | G | A |
| 17 | 46669430 | rs9299 | T | C |
| 17 | 80052073 | rs9905991 | A | G |
| 18 | 42950629 | rs10438964 | T | C |
| 18 | 73498528 | rs11150911 | A | C |
| 18 | 57726627 | rs12327272 | A | G |
| 18 | 6873954 | rs1241986 | G | A |
| 18 | 21116998 | rs12964689 | G | A |
| 18 | 36182440 | rs1365466 | T | C |
| 18 | 51478026 | rs1498139 | A | C |
| 18 | 947954 | rs1608445 | G | A |
| 18 | 31581247 | rs16965062 | T | C |
| 18 | 76742544 | rs1787267 | G | C |
| 18 | 37103550 | rs1791253 | T | G |
| 18 | 31251276 | rs1941697 | A | G |
| 18 | 57741783 | rs1942866 | C | G |
| 18 | 57677294 | rs2000746 | G | A |
| 18 | 63297672 | rs2012927 | A | G |
| 18 | 58039276 | rs2229616 | C | T |
| 18 | 23178748 | rs273697 | G | A |
| 18 | 40992698 | rs555267 | G | T |
| 18 | 57838401 | rs663129 | A | G |
| 18 | 45921214 | rs7239114 | A | G |
| 18 | 56883319 | rs7243357 | T | G |
| 18 | 58371566 | rs8087550 | A | C |
| 18 | 69224478 | rs8089514 | A | T |
| 18 | 52479487 | rs8092503 | G | A |
| 18 | 57804346 | rs8095404 | T | A |
| 18 | 42598463 | rs954018 | G | A |
| 18 | 57969244 | rs9675376 | A | G |
| 18 | 60739250 | rs9951893 | C | T |
| 18 | 57853056 | rs9961813 | A | C |
| 19 | 33963766 | rs10408013 | T | C |
| 19 | 46180184 | rs11672660 | C | T |
| 19 | 12994140 | rs12609744 | C | T |
| 19 | 18454825 | rs17724992 | A | G |
| 19 | 45395619 | rs2075650 | G | A |
| 19 | 19789528 | rs2304130 | A | G |
| 19 | 18215247 | rs273504 | G | A |
| 19 | 30683879 | rs2866816 | T | C |
| 19 | 34311481 | rs29938 | C | T |
| 19 | 47569003 | rs3810291 | A | G |
| 19 | 42637232 | rs3826705 | C | T |
| 19 | 30296853 | rs8102137 | C | T |
| 20 | 32738612 | rs1015362 | T | C |
| 20 | 32738335 | rs1015363 | A | G |
| 20 | 15801600 | rs12480713 | T | C |
| 20 | 62380542 | rs12625413 | C | T |
| 20 | 32542814 | rs13041173 | G | A |
| 20 | 34025756 | rs143384 | A | G |
| 20 | 32606299 | rs17091470 | G | T |
| 20 | 51107290 | rs17806379 | C | T |
| 20 | 1410582 | rs1884389 | T | C |
| 20 | 6612832 | rs1884897 | G | A |
| 20 | 41987392 | rs2143253 | G | A |
| 20 | 25971327 | rs2386802 | C | A |
| 20 | 44914134 | rs2425857 | A | G |
| 20 | 31097877 | rs293566 | T | C |
| 20 | 62127121 | rs310618 | C | T |
| 20 | 33703607 | rs3746429 | T | C |
| 20 | 16564210 | rs4814512 | A | C |
| 20 | 54157497 | rs559267 | G | A |
| 20 | 61530915 | rs6011457 | T | A |
| 20 | 47495560 | rs6019482 | C | T |
| 20 | 33594226 | rs6060151 | G | T |
| 20 | 33170752 | rs6088529 | A | C |
| 20 | 30785593 | rs6121381 | T | A |
| 20 | 26073030 | rs6132918 | C | T |
| 20 | 25059442 | rs6138482 | T | C |
| 20 | 32686658 | rs6142096 | A | G |
| 20 | 3026069 | rs676749 | A | T |
| 20 | 30504530 | rs8121840 | A | G |
| 20 | 15819495 | rs8123881 | G | A |
| 20 | 62522315 | rs8567 | G | A |
| 21 | 40309436 | rs13047416 | C | G |
| 21 | 40627020 | rs2836961 | C | A |
| 21 | 42653567 | rs2838006 | C | T |
| 21 | 46570896 | rs427943 | C | A |
| 21 | 39238610 | rs762147 | G | A |
| 21 | 34153330 | rs9979651 | G | C |
| 22 | 40604945 | rs4820408 | T | G |
| 22 | 40640285 | rs5750913 | G | A |
| 22 | 48875699 | rs9615905 | T | C |

ST2: Genetic variants associated with BMI at genome-wide significance (P<5x10-8) in the GIANT consortium of up to 339,224 people of European ancestry used in the BMI GRS for our UKB analyses

| **SNP** | **Trait raising allele** | **Other allele** | **Beta** | **SE** |
| --- | --- | --- | --- | --- |
| rs1000940 | G | A | 0.0192 | 0.0034 |
| rs10132280 | C | A | 0.023 | 0.0034 |
| rs1016287 | T | C | 0.0229 | 0.0034 |
| rs10182181 | G | A | 0.0307 | 0.0031 |
| rs10733682 | A | G | 0.0174 | 0.0031 |
| rs10938397 | G | A | 0.0402 | 0.0031 |
| rs10968576 | G | A | 0.0249 | 0.0033 |
| rs11057405 | G | A | 0.0307 | 0.0055 |
| rs11126666 | A | G | 0.0207 | 0.0034 |
| rs11165643 | T | C | 0.0218 | 0.0031 |
| rs11191560 | C | T | 0.0308 | 0.0053 |
| rs11583200 | C | T | 0.0177 | 0.0031 |
| rs1167827 | G | A | 0.0202 | 0.0033 |
| rs11688816 | G | A | 0.0172 | 0.0031 |
| rs11727676 | T | C | 0.0358 | 0.0064 |
| rs11847697 | T | C | 0.0492 | 0.0084 |
| rs12286929 | G | A | 0.0217 | 0.0031 |
| rs12401738 | A | G | 0.0211 | 0.0033 |
| rs12429545 | A | G | 0.0334 | 0.0047 |
| rs12446632 | G | A | 0.0403 | 0.0046 |
| rs12566985 | G | A | 0.0242 | 0.0031 |
| rs12885454 | C | A | 0.0207 | 0.0033 |
| rs12940622 | G | A | 0.0182 | 0.0031 |
| rs13021737 | G | A | 0.0601 | 0.004 |
| rs13078960 | G | T | 0.0297 | 0.0039 |
| rs13191362 | A | G | 0.0277 | 0.0048 |
| rs1516725 | C | T | 0.0451 | 0.0046 |
| rs1528435 | T | C | 0.0178 | 0.0031 |
| rs1558902 | A | T | 0.0818 | 0.0031 |
| rs16851483 | T | G | 0.0483 | 0.0077 |
| rs16951275 | T | C | 0.0311 | 0.0037 |
| rs17001654 | G | C | 0.0306 | 0.0053 |
| rs17024393 | C | T | 0.0658 | 0.0088 |
| rs17094222 | C | T | 0.0249 | 0.0038 |
| rs17405819 | T | C | 0.0224 | 0.0033 |
| rs17724992 | A | G | 0.0194 | 0.0035 |
| rs1808579 | C | T | 0.0167 | 0.0031 |
| rs1928295 | T | C | 0.0188 | 0.0031 |
| rs2033529 | G | A | 0.019 | 0.0033 |
| rs2033732 | C | T | 0.0192 | 0.0035 |
| rs205262 | G | A | 0.0221 | 0.0035 |
| rs2075650 | A | G | 0.0258 | 0.0045 |
| rs2112347 | T | G | 0.0261 | 0.0031 |
| rs2121279 | T | C | 0.0245 | 0.0044 |
| rs2176598 | T | C | 0.0198 | 0.0036 |
| rs2207139 | G | A | 0.0447 | 0.004 |
| rs2245368 | C | T | 0.0317 | 0.0057 |
| rs2287019 | C | T | 0.036 | 0.0042 |
| rs2365389 | C | T | 0.02 | 0.0031 |
| rs2650492 | A | G | 0.0207 | 0.0035 |
| rs2820292 | C | A | 0.0195 | 0.0031 |
| rs29941 | G | A | 0.0182 | 0.0033 |
| rs3101336 | C | T | 0.0334 | 0.0031 |
| rs3736485 | A | G | 0.0176 | 0.0031 |
| rs3810291 | A | G | 0.0283 | 0.0036 |
| rs3817334 | T | C | 0.0262 | 0.0031 |
| rs3849570 | A | C | 0.0188 | 0.0034 |
| rs4256980 | G | C | 0.0209 | 0.0031 |
| rs4740619 | T | C | 0.0179 | 0.0031 |
| rs543874 | G | A | 0.0482 | 0.0039 |
| rs6477694 | C | T | 0.0174 | 0.0031 |
| rs6567160 | C | T | 0.0556 | 0.0036 |
| rs657452 | A | G | 0.0227 | 0.0031 |
| rs6804842 | G | A | 0.0185 | 0.0031 |
| rs7138803 | A | G | 0.0315 | 0.0031 |
| rs7141420 | T | C | 0.0235 | 0.0031 |
| rs7243357 | T | G | 0.0217 | 0.004 |
| rs758747 | T | C | 0.0225 | 0.0037 |
| rs7599312 | G | A | 0.022 | 0.0034 |
| rs7899106 | G | A | 0.0395 | 0.0071 |
| rs9581854 | T | C | 0.03 | 0.005 |
| rs9400239 | C | T | 0.0188 | 0.0033 |
| rs9925964 | A | G | 0.0192 | 0.0031 |

ST3: A table showing our health satisfaction outcome in CKB stratified by sex and region of residence within China.

| **Region** | **Urban_Rural** | **Sex** | **0 – Poor** | **1 – Fair** | **2 – Good** | **3 – Excellent** |
| --- | --- | --- | --- | --- | --- | --- |
| Qingdao | Urban | Female N(%) | 331 (6.7) | 2486 (50.0) | 1162 (23.4) | 995 (20.0) |
|  |  | Male N(%) | 198 (6.0) | 1,428 (43.4) | 941 (28.6) | 720 (21.9) |
| Harbin | Urban | Female N(%) | 987 (12.8) | 3303 (42.8) | 1340 (17.3) | 2097 (27.1) |
|  |  | Male N(%) | 530 (9.8) | 2056 (38.1) | 1052 (19.5) | 1756 (32.6) |
| Haikou | Urban | Female N(%) | 305 (8.5) | 2173 (60.2) | 695 (19.3) | 434 (12.0) |
|  |  | Male N(%) | 165 (7.6) | 1182 (54.2) | 508 (23.3) | 325 (14.9) |
| Suzhou | Urban | Female N(%) | 593 (12.9) | 1261 (27.5) | 1539 (33.6) | 1194 (26.0) |
|  |  | Male N(%) | 304 (9.0) | 824 (24.3) | 1138 (33.6) | 1124 (33.2) |
| Liuzhou | Urban | Female N(%) | 472 (9.3) | 2939 (58.1) | 947 (18.7) | 703 (13.9) |
|  |  | Male N(%) | 381 (10.2) | 1977 (53.0) | 730 (19.6) | 644 (17.3) |
| Sichuan | Rural | Female N(%) | 1701 (26.8) | 2570 (40.5) | 1720 (27.1) | 356 (5.6) |
|  |  | Male N(%) | 870 (20.3) | 1785 (41.7) | 1298 (30.3) | 331 (7.7) |
| Gansu | Rural | Female N(%) | 886 (15.0) | 2401 (40.7) | 1674 (28.4) | 932 (15.8) |
|  |  | Male N(%) | 444 (10.7) | 1455 (35.0) | 1192 (28.7) | 1069 (25.7) |
| Henan | Rural | Female N(%) | 981 (16.3) | 2596 (43.0) | 1807 (30.0) | 650 (10.9) |
|  |  | Male N(%) | 686 (12.8) | 2216 (41.2) | 1725 (32.1) | 752 (14.0) |
| Zhejiang | Rural | Female N(%) | 422 (6.1) | 2567 (37.3) | 3017 (43.8) | 877 (12.7) |
|  |  | Male N(%) | 256 (5.0) | 2015 (39.1) | 2108 (40.9) | 771 (15.0) |
| Hunan | Rural | Female N(%) | 571 (8.8) | 3891 (60.2) | 1275 (19.7) | 723 (11.2) |
|  |  | Male N(%) | 533(8.8) | 3459 (57.2) | 1319 (21.8) | 734 (12.1) |

ST4: A table showing our life satisfaction outcome in CKB stratified by sex and region of residence within China.

| **Region** | **Urban_Rural** | **Sex** | **0 – Unsatisfied** | **1 – Neither** | **2 – Satisfied** | **3 - Very satisfied** |
| --- | --- | --- | --- | --- | --- | --- |
| Qingdao | Urban | Female N(%) | 108 (2.2) | 1,445 (29.1) | 2633 (52.9) | 788 (15.8) |
|  |  | Male N(%) | 127 (3.9) | 954 (29.0) | 1700 (51.7) | 506 (15.4) |
| Harbin | Urban | Female N(%) | 415 (5.4) | 1213 (5.7) | 5144 (66.6) | 955 (12.4) |
|  |  | Male N(%) | 344(6.4) | 734 (13.6) | 3594 (66.6) | 722 (13.4) |
| Haikou | Urban | Female N(%) | 250 (6.9) | 1939 (53.8) | 1333 (37.0) | 85 (2.4) |
|  |  | Male N(%) | 157 (7.2) | 1020 (46.8) | 925 (42.4) | 78 (3.6) |
| Suzhou | Urban | Female N(%) | 449 (9.8) | 1,646 (35.9) | 1,826 (39.8) | 666 (14.5) |
|  |  | Male N(%) | 269 (7.9) | 1,094 (32.3) | 1,587 (46.8) | 440 (13.0) |
| Liuzhou | Urban | Female N(%) | 375 (7.4) | 1,868 (36.9) | 2,559 (50.6) | 259 (5.1) |
|  |  | Male N(%) | 357 (9.6) | 1,282 (34.4) | 1,916 (51.3) | 177 (4.7) |
| Sichuan | Rural | Female N(%) | 194 (3.1) | 1,957 (30.8) | 2,981 (47.0) | 1,215 (19.1) |
|  |  | Male N(%) | 108 (2.5) | 1,033 (24.1) | 2,245 (52.4) | 898 (21.0) |
| Gansu | Rural | Female N(%) | 133 (2.3) | 484 (8.2) | 2,937 (49.8) | 2,339 (39.7) |
|  |  | Male N(%) | 134 (3.2) | 326 (7.8) | 1,980 (47.6) | 1,720 (41.4) |
| Henan | Rural | Female N(%) | 193 (3.2) | 1,180 (19.6) | 1,733 (28.7) | 2,928 (48.5) |
|  |  | Male N(%) | 239 (4.4) | 881 (16.4) | 1,847 (34.3) | 2,412 (44.8) |
| Zhejiang | Rural | Female N(%) | 251 (3.7) | 2,486 (36.1) | 3,619 (52.6) | 527 (7.7) |
|  |  | Male N(%) | 173 (3.4) | 1,975 (38.4) | 2,685 (52.1) | 317 (6.2) |
| Hunan | Rural | Female N(%) | 45 (0.7) | 2,276 (35.2) | 3,842 (59.5) | 297 (4.6) |
|  |  | Male N(%) | 55 (0.9) | 2,051 (33.9) | 3,674 (60.8) | 265 (4.4) |

ST5: The observational associations between BMI and health and life satisfaction in the CKB and UKB stratified by urban and rural regions further adjusting for measures of socioeconomic status.

|  |  |  | **CKB** | | **UKB** | |
| --- | --- | --- | --- | --- | --- | --- |
| **Study** | **Strata** | **Region** | **Beta (95% CI) per SD higher BMI** | **Pa** | **Beta (95% CI) per SD higher BMI** | **Pb** |
| Health satisfaction | All | Both | 0.020 (0.015, 0.026) | 3.30E-12 | -0.162 (-0.164, -0.159) | <1.00E-15 |
|  | Male | Both | 0.028 (0.018, 0.037) | 2.60E-09 | -0.171 (-0.175, -0.167) | <1.00E-15 |
|  | Female | Both | 0.018 (0.010, 0.025) | 2.50E-06 | -0.154 (-0.158, -0.150) | <1.00E-15 |
|  | All | Urban only | 0.011 (0.001, 0.020) | 0.02 | -0.161 (-0.164, -0.158) | <1.00E-15 |
|  | Male | Urban only | 0.012 (-0.003, 0.027) | 0.11 | -0.170 (-0.174, -0.166) | <1.00E-15 |
|  | Female | Urban only | 0.010 (-0.002, 0.022) | 0.09 | -0.154 (-0.158, -0.150) | <1.00E-15 |
|  | All | Rural only | 0.025 (0.017, 0.032) | 4.70E-11 | -0.162 (-0.169, -0.155) | <1.00E-15 |
|  | Male | Rural only | 0.037 (0.026, 0.049) | 2.30E-10 | -0.175 (-0.185, -0.165) | <1.00E-15 |
|  | Female | Rural only | 0.019 (0.009, 0.028) | 1.10E-04 | -0.151 (-0.160, -0.141) | <1.00E-15 |
| Life Satisfaction | All | Both | 0.054 (0.049, 0.059) | <1.00E-15 | -0.008 (-0.013, -0.003) | 3.20E-03 |
|  | Male | Both | 0.048 (0.040, 0.056) | <1.00E-15 | 0.000 (-0.007, 0.008) | 0.92 |
|  | Female | Both | 0.060 (0.053, 0.066) | <1.00E-15 | -0.014 (-0.021, -0.006) | 2.30E-04 |
|  | All | Urban only | 0.031 (0.024, 0.038) | <1.00E-15 | -0.008 (-0.014, -0.002) | 5.60E-03 |
|  | Male | Urban only | 0.025 (0.014, 0.036) | <1.00E-15 | -0.002 (-0.011, 0.006) | 0.61 |
|  | Female | Urban only | 0.036 (0.027, 0.045) | 3.80E-15 | -0.012 (-0.020, -0.004) | 3.10E-03 |
|  | All | Rural only | 0.059 (0.053, 0.066) | <1.00E-15 | -0.008 (-0.022, 0.005) | 0.21 |
|  | Male | Rural only | 0.060 (0.050, 0.070) | <1.00E-15 | 0.013 (-0.006, 0.032) | 0.18 |
|  | Female | Rural only | 0.057 (0.049, 0.066) | <1.00E-15 | -0.024 (-0.042, -0.006) | 0.01 |

Pa adjusted for age, region, sex (all only), SES, smoking, alcohol consumption, education and household income.

Pb adjusted for age, centre, sex (all only), TDI, smoking, alcohol consumption, education and household income.

ST6: The comparison of the 1-sample genetic associations between BMI and health satisfaction in the CKB and the UKB.

|  |  | **CKB** | **UKB** |  |
| --- | --- | --- | --- | --- |
| **Strata** | **Region** | **Beta (SE)** | **Beta (SE)** | **P_difference_** |
| All | Both | 0.026 (0.017) | -0.183 (0.009) | <1.00E-15 |
| Male | Both | -0.006 (0.030) | -0.231 (0.012) | 1.75E-12 |
| Female | Both | 0.041 (0.020) | -0.206 (0.012) | 1.24E-04 |
| All | Urban only | 0.022 (0.027) | -0.184 (0.010) | 2.79E-04 |
| Male | Urban only | -0.004 (0.049) | -0.234 (0.013) | 4.98E-06 |
| Female | Urban only | 0.035 (0.033) | -0.203 (0.012) | 1.23E-11 |
| All | Rural only | 0.028 (0.021) | -0.167 (0.023) | 5.37E-10 |
| Male | Rural only | -0.008 (0.037) | -0.173 (0.031) | 6.01E-04 |
| Female | Rural only | 0.045 (0.026) | -0.177 (0.032) | 6.75E-08 |

P_difference_ calculated using Fishers z-score method.

ST7: The meta-analysed estimates with heterogeneity statistics for genetic 1-sample associations between BMI and health and life satisfaction in individuals with valid genetic data from the China Kadoorie Biobank .

| **Outcome** | **Sex** | **Region** | **beta (95% CI)** | **P^a^** | **Cochran's Q** | **Heterogeneity statistic** | **P_het_** | | **I^2^** |
| --- | --- | --- | --- | --- | --- | --- | --- | --- | --- |
| Health Satisfaction | All | Both | 0.026 (-0.007, 0.059) | 0.117 | 65.76 | 1.860 | <1.00E-15 | | 71.10% |
|  | Male | Both | -0.006 (-0.064, 0.052) | 0.831 | 23.51 | 1.616 | 0.005 | | 61.70% |
|  | Female | Both | 0.041 (0.002, 0.081) | 0.041 | 40.48 | 2.121 | <1.00E-15 | | 77.80% |
|  | All | Urban only | 0.022 (-0.031, 0.076) | 0.412 | 26.81 | 1.726 | 0.002 | | 66.40% |
|  | Male | Urban only | -0.004 (-0.100, 0.091) | 0.932 | 10.49 | 1.619 | 0.033 | | 61.90% |
|  | Female | Urban only | 0.035 (-0.030, 0.099) | 0.295 | 15.89 | 1.993 | 0.003 | | 74.80% |
|  | All | Rural only | 0.028 (-0.013, 0.070) | 0.178 | 38.92 | 2.080 | <1.00E-15 | | 76.90% |
|  | Male | Rural only | -0.008 (-0.080, 0.065) | 0.839 | 13.02 | 1.804 | 0.011 | | 69.30% |
|  | Female | Rural only | 0.045 (-0.005, 0.095) | 0.076 | 24.52 | 2.476 | <1.00E-15 | | 83.70% |
| Life Satisfaction | All | Both | -0.028 (-0.055, -0.002) | 0.038 | 43.34 | 1.510 | 0.001 | | 56.20% |
|  | Male | Both | -0.077 (-0.123, -0.030) | 0.001 | 16.37 | 1.349 | 0.060 | | 45.00% |
|  | Female | Both | -0.004 (-0.037, 0.028) | 0.797 | 20.66 | 1.515 | 0.014 | | 56.40% |
|  | All | Urban only | 0.006 (-0.036, 0.048) | 0.784 | 14.95 | 1.289 | 0.092 | | 39.80% |
|  | Male | Urban only | -0.033 (-0.109, 0.042) | 0.388 | 6.24 | 1.249 | 0.182 | | 35.90% |
|  | Female | Urban only | 0.024 (-0.027, 0.075) | 0.360 | 7.20 | 1.341 | 0.126 | | 44.40% |
|  | All | Rural only | -0.051 (-0.085, -0.016) | 0.004 | 24.24 | 1.641 | 0.004 | | 62.90% |
|  | Male | Rural only | -0.103 (-0.162, -0.044) | 0.001 | 8.07 | 1.420 | 0.089 | | 50.40% |
|  | Female | Rural only | -0.023 (-0.066, 0.019) | 0.275 | 11.50 | 1.696 | 0.021 | | 65.20% |
| Life satisfaction removing Hunan with prevalence unsatisfied <1% | All | Both | 0.000 (-0.030, 0.029) | 0.974 | 24.93 | 1.211 | 0.096 | | 31.80% |
|  | Male | Both | -0.043 (-0.096, 0.010) | 0.114 | 9.64 | 1.098 | 0.291 | 17.00% | |
|  | Female | Both | 0.019 (-0.017, 0.055) | 0.302 | 11.73 | 1.211 | 0.164 | 31.80% | |
|  | All | Urban only | 0.006 (-0.036, 0.048) | 0.784 | 14.95 | 1.289 | 0.092 | 39.80% | |
|  | Male | Urban only | -0.033 (-0.109, 0.042) | 0.388 | 6.24 | 1.249 | 0.182 | 35.90% | |
|  | Female | Urban only | 0.024 (-0.027, 0.075) | 0.360 | 7.20 | 1.341 | 0.126 | 44.40% | |
|  | All | Rural only | -0.007 (-0.048, 0.035) | 0.751 | 9.81 | 1.184 | 0.199 | 28.70% | |
|  | Male | Rural only | -0.052 (-0.126, 0.022) | 0.171 | 3.28 | 1.045 | 0.351 | 8.50% | |
|  | Female | Rural only | 0.014 (-0.036, 0.064) | 0.586 | 4.46 | 1.219 | 0.216 | 32.70% | |

*P^a^ adjusted for age, regional principal components and chip*

ST8: The comparison of the 1-sample genetic associations between BMI and life satisfaction in the CKB and the UKB.

|  |  | **CKB** | **UKB** |  |  |
| --- | --- | --- | --- | --- | --- |
| **Strata** | **Region** | **Beta (SE)** | **Beta (SE)** | **P_difference_** | |
| All | Both | -0.028 (0.014) | -0.019 (0.018) | 0.69 | |
| Male | Both | -0.077 (0.024) | -0.003 (0.024) | 0.03 | |
| Female | Both | -0.004 (0.017) | -0.030 (0.022) | 0.36 | |
| All | Urban only | 0.006 (0.021) | -0.027 (0.02) | 0.25 | |
| Male | Urban only | -0.033 (0.039) | -0.003 (0.026) | 0.52 | |
| Female | Urban only | 0.024 (0.026) | -0.031 (0.024) | 0.12 | |
| All | Rural only | -0.051 (0.018) | 0.030 (0.045) | 0.09 | |
| Male | Rural only | -0.103 (0.030) | 0.026 (0.057) | 0.04 | |
| Female | Rural only | -0.023 (0.022) | 0.010 (0.062) | 0.62 | |

P_difference_ calculated using Fishers z-score method.

ST9: The genetic 1-sample associations between BMI and life satisfaction in the CKB removing Hunan stratified by sex and region.

|  |  | **Genetic** | |
| --- | --- | --- | --- |
| **Strata** | **Region** | **beta (95% CI) per SD higher BMI** | **Pa** |
| All | Both | 0.000 (-0.030, 0.029) | 0.97 |
| Male | Both | -0.043 (-0.096, 0.010) | 0.11 |
| Female | Both | 0.019 (-0.017, 0.055) | 0.30 |
| All | Urban only | 0.006 (-0.036, 0.048) | 0.78 |
| Male | Urban only | -0.033 (-0.109, 0.042) | 0.39 |
| Female | Urban only | 0.024 (-0.027, 0.075) | 0.36 |
| All | Rural only | -0.007 (-0.048, 0.035) | 0.75 |
| Male | Rural only | -0.052 (-0.126, 0.022) | 0.17 |
| Female | Rural only | 0.014 (-0.036, 0.064) | 0.59 |

*Pa adjusted for age, region, sex and principal components*

ST10:Linear, Quadratic and Fractional Polynomial models to predict health satisfaction from BMI in the CKB and the UKB. The deviance difference compares the fit with that of a straight line (p=1). Here, we use the AIC to determine which model provides a better fit (lower AIC = better fit).

| **Study** | **Region** | **Strata** | **FP1 power** | **Deviance FP1** | **FP1 AIC** | **Pa** | **FP2 powers** | **Deviance FP2** | **FP2 AIC** | **Pb** | **Pc** | **Quadratic AIC** | **Linear AIC** |
| --- | --- | --- | --- | --- | --- | --- | --- | --- | --- | --- | --- | --- | --- |
| UKB | Both | All | 2 | 793873.44 | 793923.4 | <0.001 | -2, -2 | 791515.26 | **791567.3** | <0.001 | <0.001 | 793925.4 | 794533.8 |
| UKB | Both | Male | 2 | 375915.3 | 375963.3 | <0.001 | -2, -2 | 374413.16 | **374463.2** | <0.001 | <0.001 | 375908 | 376425.8 |
| UKB | Both | Female | 2 | 417027.14 | 417075.1 | <0.001 | -2, -2 | 415919.52 | **415919.5** | <0.001 | <0.001 | 417053.3 | 417258.5 |
| CKB | Both | All | -2 | 259512.68 | 259538.7 | <0.001 | -0.5, -0.5 | 259138.13 | **259166.1** | <0.001 | <0.001 | 259246.1 | 259697.3 |
| CKB | Both | Male | -2 | 111518.2 | 111518.2 | <0.001 | -0.5, -0.5 | 111326.15 | **111326.2** | <0.001 | <0.001 | 111377.3 | 111617.2 |
| CKB | Both | Female | -2 | 147647.5 | 147841.7 | <0.001 | -0.5, -0.5 | 147621.55 | **147647.5** | <0.001 | <0.001 | 147691.1 | 147908.6 |
| UKB | Urban only | All | 2 | 672920.81 | 672970.8 | <0.001 | -2, -2 | 670860.74 | **670912.7** | <0.001 | <0.001 | 672972.5 | 673507.2 |
| UKB | Urban only | Male | 2 | 319130.8 | 319178.8 | <0.001 | -2, -2 | 317838.37 | **317888.4** | <0.001 | <0.001 | 319119.8 | 319597.3 |
| UKB | Urban only | Female | 2 | 352961.87 | 353009.9 | <0.001 | -2, -2 | 351981.55 | **352031.6** | <0.001 | <0.001 | 352991.1 | 353161.2 |
| CKB | Urban only | All | -2 | 117850.54 | 117866.5 | <0.001 | -1, -0.5 | 117682.41 | **117700.4** | <0.001 | <0.001 | 117739.1 | 117904 |
| CKB | Urban only | Male | -2 | 48514.17 | 48528.17 | <0.001 | -0.5, 0 | 48445.38 | **48461.38** | <0.001 | <0.001 | 48468.99 | 48552.08 |
| CKB | Urban only | Female | -2 | 69295.98 | 69309.98 | <0.001 | -1, -1 | 69196.74 | **69212.74** | <0.001 | <0.001 | 69240.05 | 69324.53 |
| UKB | Rural only | All | 2 | 112250.31 | 112300.3 | <0.001 | -2, -2 | 112025.04 | **112077** | <0.001 | <0.001 | 112301.3 | 112362.8 |
| UKB | Rural only | Male | 2 | 52237.77 | 52283.77 | <0.001 | -2, -2 | 52071.38 | **52119.38** | <0.001 | <0.001 | 52285.77 | 52313.86 |
| UKB | Rural only | Female | 2 | 59894.59 | 59942.59 | <0.001 | -2, -1 | 59809.46 | **59859.46** | <0.001 | <0.001 | 59943.07 | 59975.16 |
| CKB | Rural only | All | -2 | 141168.31 | 141184.3 | <0.001 | -0.5, -0.5 | 140971.53 | **140989.5** | <0.001 | <0.001 | 141028.2 | 141293.4 |
| CKB | Rural only | Male | -2 | 62773.49 | 62787.49 | <0.001 | -1, -0.5 | 62682.13 | **62698.13** | <0.001 | <0.001 | 62714.69 | 62855.62 |
| CKB | Rural only | Female | -2 | 78215.81 | 78229.81 | <0.001 | -0.5, -0.5 | 78120.72 | **78136.72** | <0.001 | <0.001 | 78153.86 | 78274.15 |

*Pa comparison of FP1 to linear*

*Pb comparison of FP2 to linear*

*Pc comparison of FP2 to FP1*

*Bold values identify the lowest AIC value and therefore the best fitting model*

ST11:Linear, Quadratic and Fractional Polynomial models to predict life satisfaction from BMI in the CKB and the UKB. The deviance difference compares the fit with that of a straight line (p=1). Here, we use the AIC to determine which model provides a better fit (lower AIC = better fit).

| **Study** | **Region** | **Strata** | **FP1 power** | **Deviance FP1** | **FP1 AIC** | **Pa** | **FP2 powers** | **Deviance FP2** | **FP2 AIC** | **Pb** | **Pc** | **Quadratic AIC** | **Linear AIC** |
| --- | --- | --- | --- | --- | --- | --- | --- | --- | --- | --- | --- | --- | --- |
| UKB | Both | All | 3 | 287242.15 | 287292.2 | <0.001 | -2, 0 | 287093.71 | **287145.7** | <0.001 | <0.001 | 287213.8 | 287370.8 |
| UKB | Both | Male | 3 | 126500.36 | 126548.4 | <0.001 | -2, 0 | 126365.95 | **126415.9** | <0.001 | <0.001 | 126452.5 | 126570.3 |
| UKB | Both | Female | 3 | 160480.82 | 160528.8 | <0.001 | -2, -2 | 160392.37 | **160442.4** | <0.001 | <0.001 | 160506.5 | 160575.2 |
| CKB | Both | All | -2 | 219393.45 | 219419.4 | <0.001 | 0, 0 | 219385.7 | **219413.7** | <0.001 | 0.021 | 219418.6 | 219486.8 |
| CKB | Both | Male | -2 | 93689.1 | 93713.1 | <0.001 | 2, 3 | 93683.15 | **93709.15** | <0.001 | 0.051 | 93709.95 | 93732.37 |
| CKB | Both | Female | -2 | 125564.26 | 125588.3 | <0.001 | -2, -1 | 125556.33 | **125582.3** | <0.001 | 0.019 | 125591.7 | 125624.8 |
| UKB | Urban only | All | 3 | 238335.62 | 238385.6 | <0.001 | -2, -0.5 | 238196.81 | **238248.8** | <0.001 | <0.001 | 238314.5 | 238457.8 |
| UKB | Urban only | Male | 3 | 105114 | 105162 | <0.001 | -2, 0 | 105002.59 | **105052.6** | <0.001 | <0.001 | 105081.8 | 105186.5 |
| UKB | Urban only | Female | 3 | 132995.01 | 133043 | <0.001 | -2, -2 | 132910.04 | **132960** | <0.001 | <0.001 | 133020.8 | 133084.8 |
| CKB | Urban only | All | -2 | 96285.53 | 96301.53 | <0.001 | -2, 2 | 96272.04 | **96290.04** | <0.001 | 0.001 | 96295.03 | 96335.38 |
| CKB | Urban only | Male | -2 | 39663.46 | 39677.46 | 0.001 | 2, 2 | 39658.73 | **39674.73** | 0.001 | 0.094 | 39675.03 | 39689.42 |
| CKB | Urban only | Female | -2 | 56555.28 | 56569.28 | <0.001 | -2, -2 | 56541.79 | **56557.79** | <0.001 | 0.001 | 56567.12 | 56588.35 |
| UKB | Rural only | All | 3 | 45704.97 | 45754.97 | 0.037 | -1, -1 | 45696.64 | **45748.64** | 0.005 | 0.016 | 45753.12 | 45759.31 |
| UKB | Rural only | Male | -2 | 19838.12 | 19884.12 | 0.070 | -2, -2 | 19828.26 | **19876.26** | 0.004 | 0.007 | 19882.95 | 19887.41 |
| UKB | Rural only | Female | 3 | 25809.92 | 25857.92 | 0.095 | -2, -2 | 25804.06 | **25854.06** | 0.035 | 0.054 | 25859.09 | 25860.71 |
| CKB | Rural only | All | -2 | 123054.8 | **123070.8** | <0.001 | -0.5, -0.5 | 123054.64 | 123072.6 | <0.001 | 0.922 | 123074.8 | 123090.4 |
| CKB | Rural only | Male | -0.5 | 53977.32 | **53991.32** | 0.080 | 1, 3 | 53977.09 | 53993.09 | 0.346 | 0.887 | 53993.12 | 53994.4 |
| CKB | Rural only | Female | -2 | 68989.26 | **69003.26** | 0.001 | -2, -2 | 68988.99 | 69004.99 | 0.007 | 0.875 | 69008.35 | 69014.96 |

Pa comparison of FP1 to linear

Pb comparison of FP2 to linear

Pc comparison of FP2 to FP1

Bold values identify the lowest AIC value and therefore the best fitting model

ST12: The non-linear MR results of BMI to health satisfaction.

|  | |  | **CKB** | | **UKB** | |  |  |
| --- | --- | --- | --- | --- | --- | --- | --- | --- |
| **Strata** | **Region** | | **Predicted quadratic beta (95% CI) per SD higher BMI** | **P^a^** | **Predicted quadratic beta (95% CI) per SD higher BMI** | **P^b^** | **P_comparison_** | |
| All | Both | | -0.042 (-0.046, -0.038) | <1.00E-15 | -0.068 (-0.069, -0.066) | <1.00E-15 | <1.00E-15 | |
| Male | Both | | -0.044 (-0.051, -0.038) | <1.00E-15 | -0.073 (-0.076, -0.071) | <1.00E-15 | <1.00E-15 | |
| Female | Both | | -0.04 (-0.046, -0.035) | <1.00E-15 | -0.065 (-0.067, -0.063) | <1.00E-15 | <1.00E-15 | |
| All | Urban only | | -0.039 (-0.046, -0.032) | <1.00E-15 | -0.068 (-0.070, -0.066) | <1.00E-15 | 2.20E-15 | |
| Male | Urban only | | -0.039 (-0.050, -0.028) | 3.98E-12 | -0.074 (-0.077, -0.071) | <1.00E-15 | 1.26E-09 | |
| Female | Urban only | | -0.038 (-0.047, -0.029) | <1.00E-15 | -0.065 (-0.068, -0.063) | <1.00E-15 | 6.68E-09 | |
| All | Rural only | | -0.044 (-0.049, -0.038) | <1.00E-15 | -0.061 (-0.065, -0.057) | <1.00E-15 | 1.82E-06 | |
| Male | Rural only | | -0.047 (-0.056, -0.039) | <1.00E-15 | -0.064 (-0.071, -0.058) | <1.00E-15 | 1.72E-03 | |
| Female | Rural only | | -0.041 (-0.048, -0.034) | <1.00E-15 | -0.061 (-0.067, -0.056) | <1.00E-15 | 8.47E-06 | |

| *P^a^ adjusted for age, regional principal components and chip* |
| --- |
| *P^b^ adjusted for age, principal components and chip* |
| *P_comparison_ of CKB and UKB estimates using Fischer's Z-score* |

ST13: The non-linear MR results of BMI to life satisfaction.

|  |  | **CKB** | | **UKB** | | |  |
| --- | --- | --- | --- | --- | --- | --- | --- |
| **Strata** | **Region** | **Predicted quadratic beta (95% CI) per SD higher BMI** | **P^a^** | **Predicted quadratic beta (95% CI) per SD higher BMI** | | **P^b^** | **P_comparison_** |
| All | Both | -0.006 (-0.009, -0.002) | 1.00E-03 | -0.031 (-0.034, -0.027) | | <1.00E-15 | <1.00E-15 |
| Male | Both | 0.001 (-0.005, 0.006) | 0.809 | -0.033 (-0.038, -0.028) | | <1.00E-15 | <1.00E-15 |
| Female | Both | -0.011 (-0.015, -0.006) | 5.73E-06 | -0.031 (-0.035, -0.027) | | <1.00E-15 | <1.00E-15 |
| All | Urban only | -0.010 (-0.016, -0.005) | 1.73E-04 | -0.032 (-0.035, -0.028) | | <1.00E-15 | 6.51E-11 |
| Male | Urban only | -0.006 (-0.015, 0.002) | 0.15 | -0.033 (-0.039, -0.028) | | <1.00E-15 | 9.00E-08 |
| Female | Urban only | -0.013 (-0.020, -0.006) | 2.33E-04 | -0.032 (-0.037, -0.027) | | <1.00E-15 | 8.62E-06 |
| All | Rural only | -0.003 (-0.007, 0.002) | 0.239 | -0.022 (-0.030, -0.013) | | 3.06E-07 | 1.04E-04 |
| Male | Rural only | 0.005 (-0.002, 0.012) | 0.150 | -0.023 (-0.036, -0.010) | | 6.33E-04 | 2.37E-04 |
| Female | Rural only | -0.009 (-0.014, -0.003) | 5.00E-03 | -0.022 (-0.033, -0.011) | | 8.95E-05 | 0.04 |
| *P^a^ adjusted for age, regional principal components and chip* | | | | |  |  |  |
| *P^b^ adjusted for age, principal components and chip* | | | | |  |  |  |
| *P_comparison_ of CKB and UKB estimates using Fischer's Z-score* | | | | |  |  |  |

Supplementary figure 1. The genetic 1-sample Mendelian Randomization estimates of BMI to health satisfaction stratified by sex and region of residence within China.


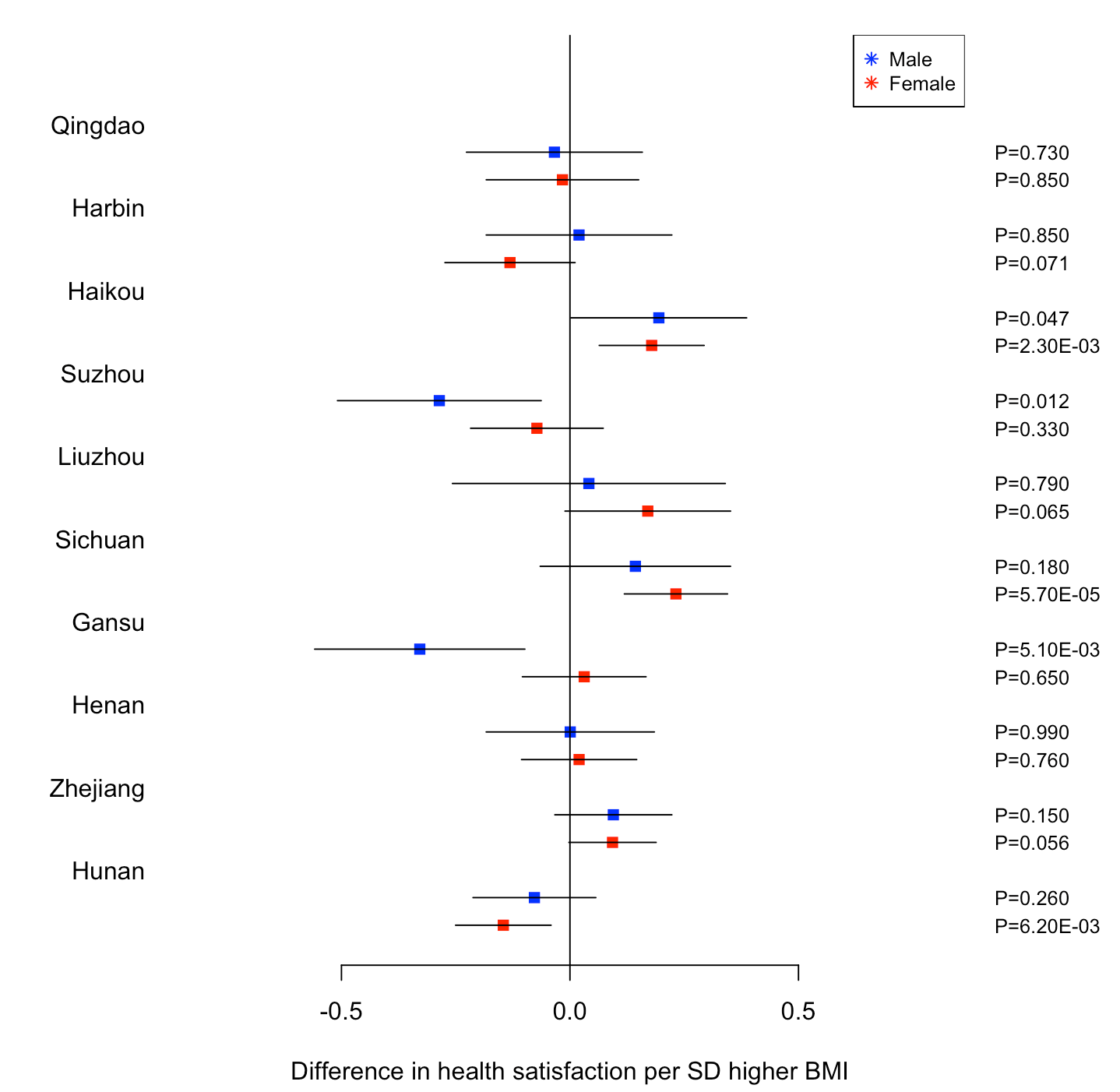


SF2: The observational and 1-sample genetic associations between BMI and life satisfaction in all individuals in the CKB and UKB, stratified by sex.


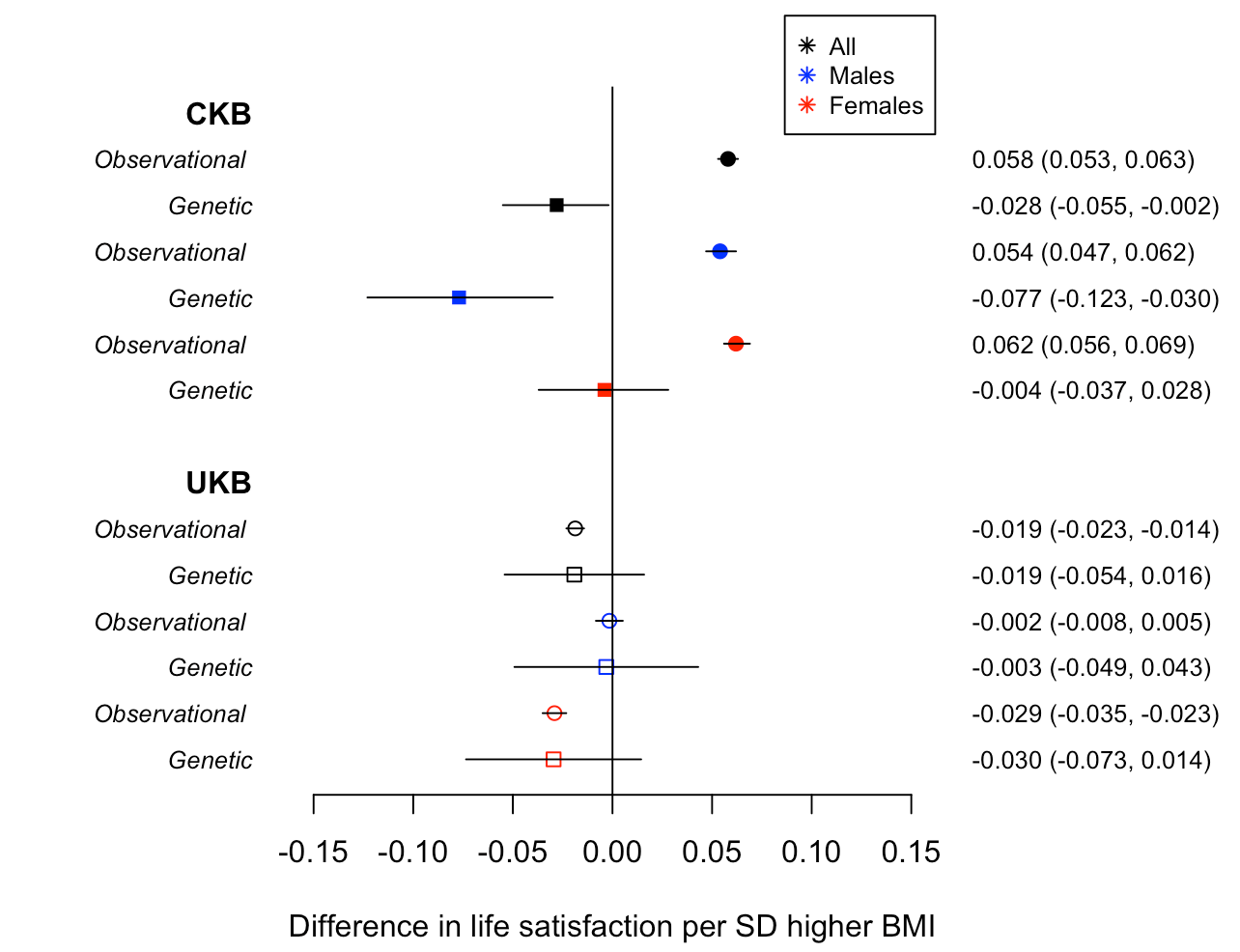


SF3: The 1-sample MR associations between BMI and life satisfaction in all individuals in the CKB and UKB stratified by sex and urban versus rural dwelling.


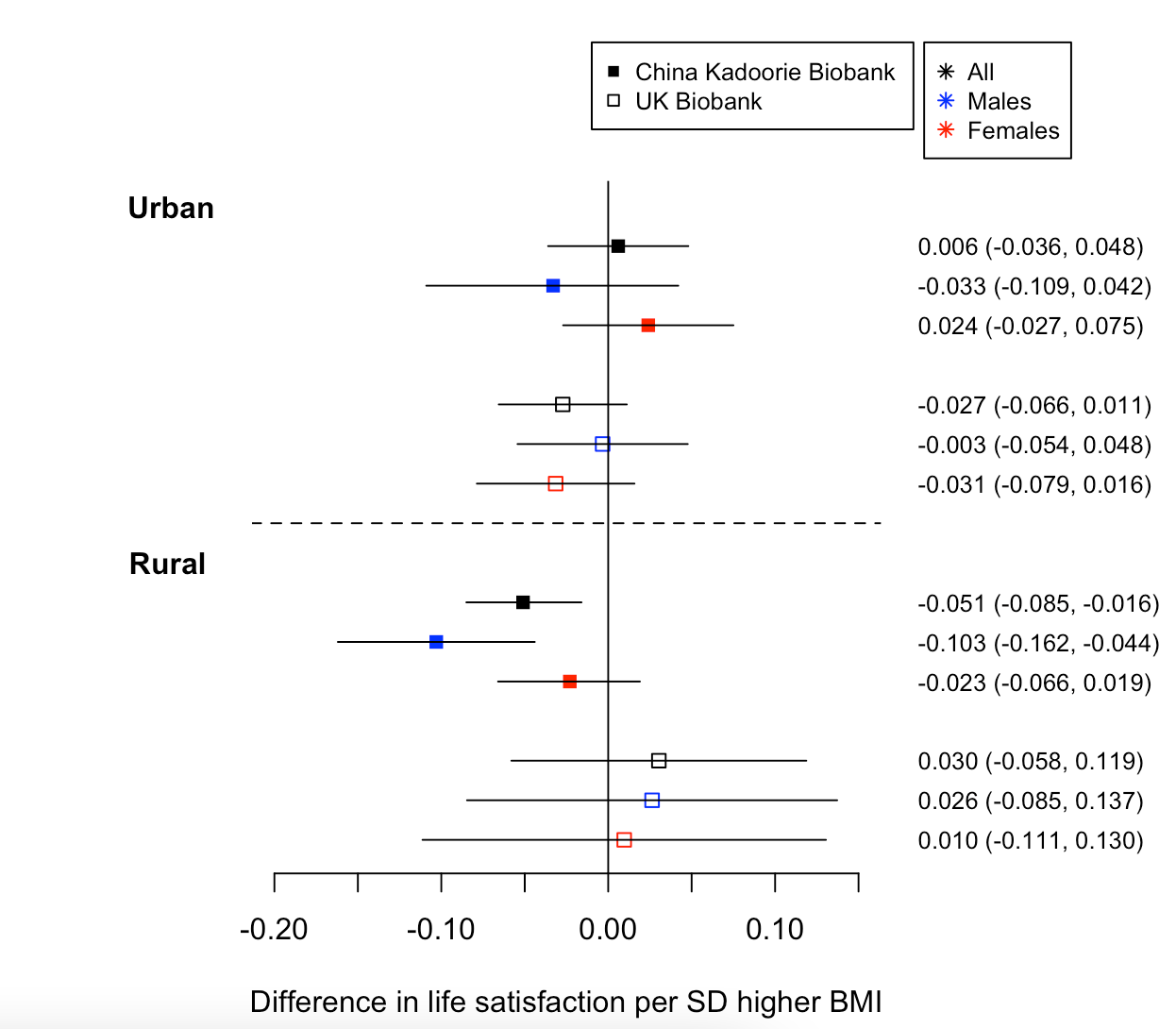


SF4: The genetic 1-sample Mendelian Randomization estimates of BMI to life satisfaction stratified by sex and region of residence within China.


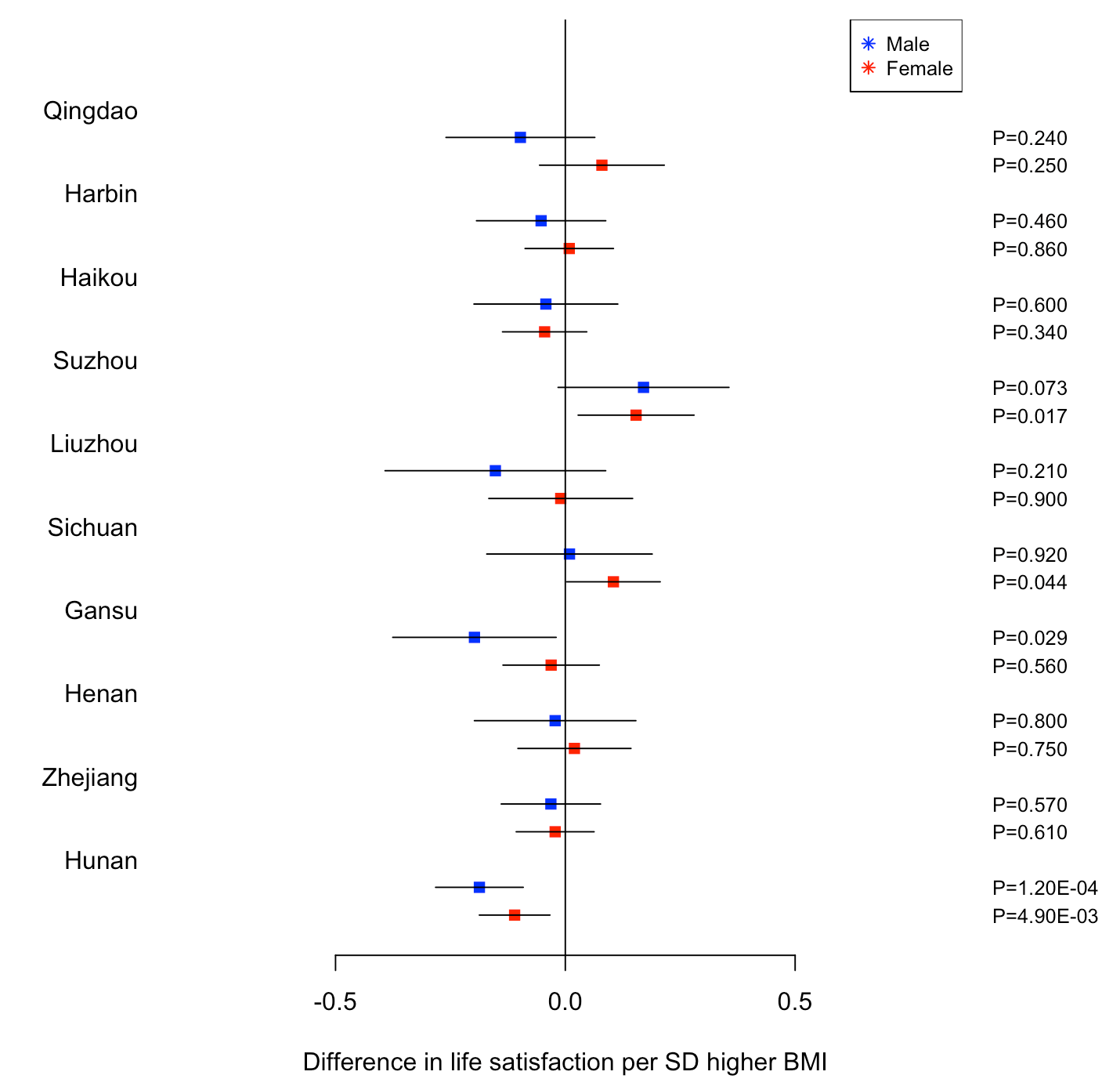


SF5: The observational and 1-sample genetic associations between BMI and life satisfaction in all individuals in the CKB and UKB, stratified by sex. CKB estimates are reported without Hunan as the prevalence of people reporting to be unsatisfied was less than 1%


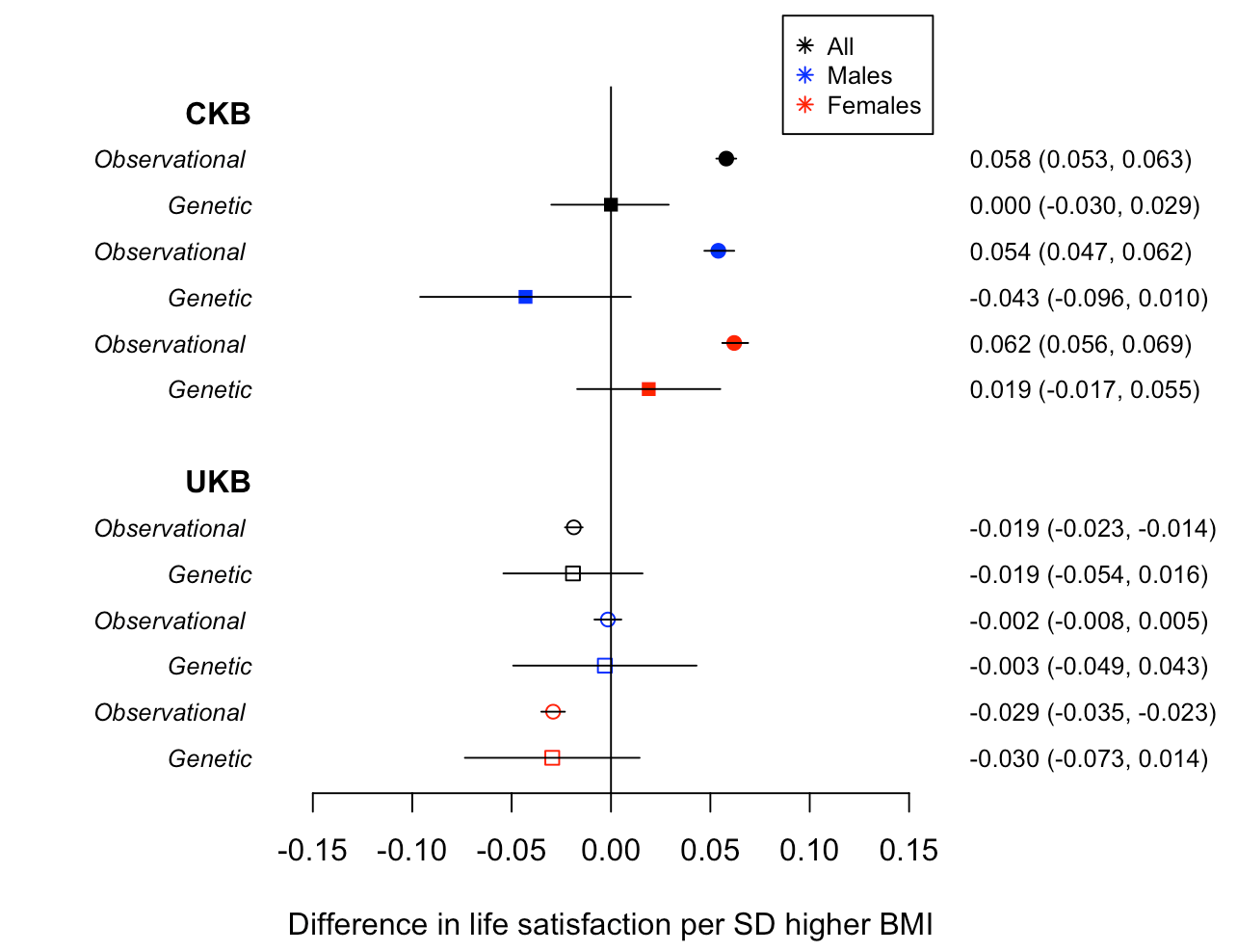


SF6: The 1-sample MR associations between BMI and life satisfaction in all individuals in the CKB and UKB stratified by sex and urban versus rural dwelling. CKB estimates are reported without Hunan as the prevalence of people reporting to be unsatisfied was less than 1%


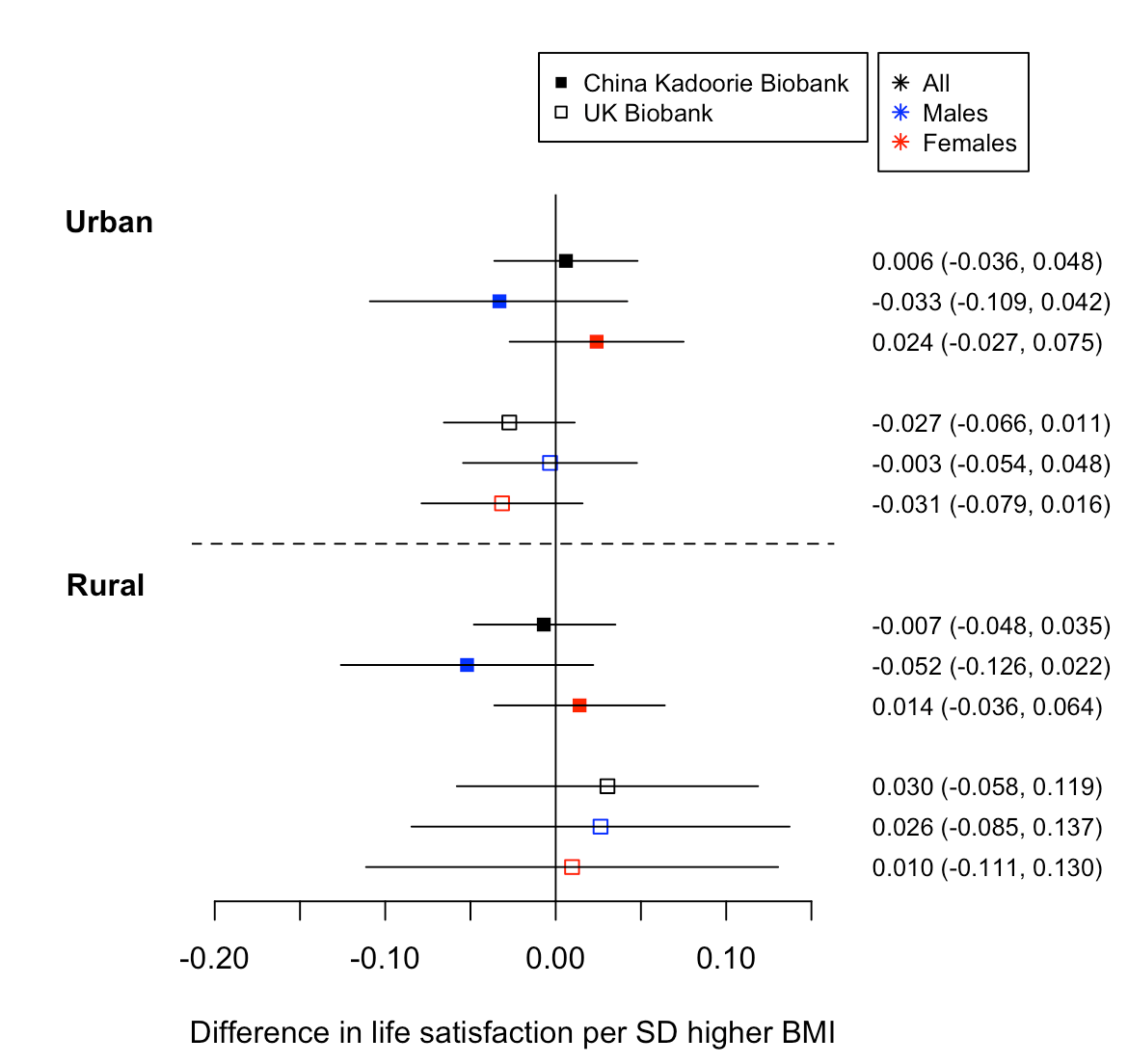

Supplement: Supplementary file 2 — Supplementary tables and figures [file 41398_2023_2539_MOESM2_ESM.docx]
